# New data on Neotropical *Scolytus* Geoffroy, 1762 with description of five new species from Peru (Coleoptera, Curculionidae, Scolytinae)

**DOI:** 10.3897/zookeys.56.519

**Published:** 2010-09-17

**Authors:** Alexander V. Petrov, Michail Y. Mandelshtam

**Affiliations:** 1 Department of Ecology and Forest Protection, Moscow State Forest University, Mytishchi-5, 141005 Moscow Region, Russia; 2 Bolshoy prospect, building 76, apt. 53, St.Petersburg, 199026, Russia

**Keywords:** bark beetles, Coleoptera, Curculionidae, new species, Peru, Scolytinae, Scolytini, Scolytus, taxonomy

## Abstract

Five new species of Scolytus Geoffroy, 1762 (Coleoptera: Curculionidae: Scolytinae) are described from Peru, namely Scolytus woodi, Scolytus carveli, Scolytus vagabundus, Scolytus lindemani, Scolytus mozolevskae .The following new synonym is established: Scolytus angustatus Browne, 1970 (= Scolytus facialis Schedl, 1973, **syn. n.**) New records of the Scolytus species in Loreto, Junin ,Cusco and Madre de Dios Provinces are given and the biology of the genus representatives is discussed.

## Introduction

An interest of entomologists to the Neotropical entomofauna studies has grown during last time. Humid tropical forests of the Amazon River basin makes researchers to amaze by the insects of unusual form and by their adaptations to live in the complex ecosystems. Scolytine (bark- and ambrosia-beetles) represent an important component of the South American xylophilous complex. The special attention to these insects is attracted by their importance for forestry. Stephen L. Wood’s monograph (2007) gave a new impulse for South American scolytine studies. By summarizing the extent knowledge on the Neotropic scolytine Wood has provided a base for further studies of this interesting group of insects.

Studies conducted recently have demonstrated the importance of the Neotropic faunal investigations, due to the fact that this region is still rich in the undescribed species new for science. Currently, many South American Scolytinae are known only from a single type specimen and knowledge of their biology and distribution so far is unknown. In some species the sex of type specimens was determined incorrectly. For example, a morphological feature of females of many Neotropic Scolytus species is the presence of specific forelocks, owing to these bunches of hairs of females accepted for males. Further entomological investigations in South America will allow making our knowledge about Scolytinae fauna and ecology more precise and will broaden our knowledge about the Scolytinae role in the xylophilous insect complexes in of the Neotropics.

In the Neotropic Region, the tribe Scolytini Latreille, 1807 is represented by genera Scolytus Geoffroy, 1762, Camptocerus Dejean, 1821, Cnemonyx Eichhoff, 1868 and Scolytopsis Blandford 1896. The tribe Scolytini attains its maximum diversity in the forests of the Central and South America. Investigations conducted during recent decades in the forests of Brasilia, Ecuador, Peru and Bolivia permitted expanding our knowledge about the species composition of Scolytini genera and about the biology of individual species in South America. New methods for insect collection allowed for the discovery and subsequent description of new species of Scolytus and Camptocerus in the Amazon River basin (Wood 2007, Petrov 2007, Smith and Cognato, in press). In the future, discovery of new taxa from the tribe Scolytini in the Neotropic Region is quite probable.

The genus Scolytus includes more than 120 species worldwide. Of these species, 37 are recorded from South America. However, paucity of collection materials prevents Scolytus species distribution range in the Neotropic Region objective analysis. However, one may already now assert that host-plants distribution is the limiting factor for the distribution of the Scolytus individual species. For the most Scolytus species, specialized oligophagy is typical, so far individual species may infest and breed in plant species from only one genus. Due to this fact, several species which breed in widely distributed lianas, possess wide distribution ranges from Mexico to Brasilia (Scolytus costatus, Scolytus cristatus), whereas the distribution of other species is limited by the basin of the Amazon River (Scolytus angustatus, Scolytus bicinctus, Scolytus amazonicus). The similarity of the orographic and climatic conditions defines the principal similarity of the plant species composition and the forest ecosystems structure in the enormous territories from North to South. So far, it was not striking to find in Peruvian forests some of the Scolytus species (Scolytus antennatus and Scolytus thoracicus) previously known from the southern Brasilia territories. Another important factor, influencing the scolytine species range, is the vertical zonality, which defines the change in plant composition in Andes. In South America at high elevations of 1800–3000 meters a.s.l. Scolytus species were not yet found. This feature is distinctive from Neotropic region fauna compared to Eurasian fauna, including Scolytus species breeding at rather high elevations.

Different methods of insect trapping were used during collecting trips. Use of specific methods were determined by landscape pecularities, forest ecosystem state and by presence of time for setting and serving of the traps. Usually barrier traps and light traps at night time are considered to be the most effective for collecting Scolytus in conditions of the South American rainforests. Nevertheless these methods do possess high efficiency, the information on biology of the species collected, namely host-specificity, peculiarities in the gallery construction etc. is lacking when these methods are used. So far, hand-collecting of beetles from the host trees is still preferential and necessary to obtain information on species biology and taxonomy.

Seventeen species from the genus Scolytus were collected during collecting trips in the provinces Loreto, Huanuco, Junin, Cusco and Madre de Dios in 1997, 2005–2009 when the humid rainforests of the Amazon, Madre de Dios, Ucayali and Urubamba river basins were visited. Most of the beetles were collected with the assistance of barrier traps.

## Systematics

### Scolytus amazonicus

Schedl, 1972

Figs 1 2 

#### Material examined.

Brazil: Manaus, Amazonas; Holotype ♂, NHMW. Peru, Loreto province, 30 km SSW from Iquitos, Panguana vill., 29.01.1997 A.Petrov (1♀).

#### Diagnosis.

The species is related to Scolytus barbatus Schedl and Scolytus mozolevskae sp.n., but can be distinguished by the structure of the second and third abdominal sternite and puncturation. The species also differs also from Scolytus barbatus by the smaller size, the absence of a bundle of golden hairs on the second abdominal sternite and also by the less abundant hair-like vestiture on the lateral parts of the front.

**Figure 1.**
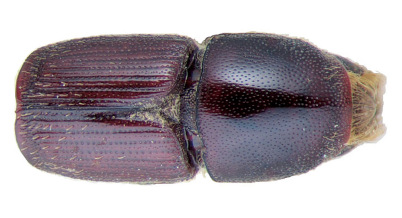
Habitus of Scolytus amazonicus, male

#### Description.

##### Male:

**Figure 2.**
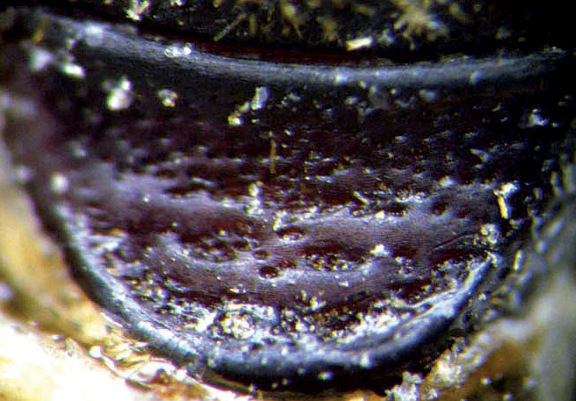
Punctation of second sternite of Scolytus amazonicus

body length 4.0–4.5 mm, 2.2–2.3 times as long as wide; body brown or reddish-brown, faintly shining, covered by pale hairs. Head reddish brown with mandibles nearly black. Front weakly convex from eye to eye and from epistoma to vertex, evenly longitudinally aciculate from vertex to epistoma. Lateral parts of front evenly rounded and covered with long golden hairs forming a brush. Antennae with reddish-brown scapus and funiculus and with blackish-brown club; club with narrow base, gradually widening towards apex, evenly rounded at apex. Pronotum 1.0–1.1 times as long as wide, reddish brown, its surface faintly shining, its punctures of variable size; punctures at base and in central portion of disk small, shallow, of elliptical form and significantly smaller compared to punctures at lateral sides in apical portion of pronotum; at base and in central part of pronotal disk punctures are sparse, in apical portion of pronotum punctures are densely set, confluent. Isolated hairs are set on lateral parts of pronotal apex. Pronotum is separated from propleura by acute lateral margin. Puncturation of lateral sides of pronotum (propleura) is inconspicuous, seen only under higher magnification, punctures are shallow and significantly smaller than punctures of pronotum lateral parts. Prothorax is covered by short, recumbent pale hairs. Scutellum triangular, set deeply in scutellar impression. Scutellum covered by minute pale scale-like hairs. Elytra 1.2–1.3 times as long as wide, equal in length to pronotum; striae distinctly, narrowly impressed, with round punctures, set close one to another; interstriae flat, with rows of small punctures. Towards elytral apex pale hairs form rows on interstriae and at lateral sides of elytra. Abdomen has first sternite set horizontal and nearly vertical second sternite. Third, fourth and fifth sternites form 45° angle with first sternite. First, fourth and fifth sternites are densely punctured with points of moderate size (Fig. 2). Puncturation of second and third sternites uneven, punctures are set on separate, slightly deepened areas and separated by dull, slightly elevated parts of surface lacking puncturation. Surface of the second and third sternites dull, the fourth glossy and shining. Legs reddish-brown. Femora with long pale hairs.

##### Female:

body length 4.0 mm, 2.3 times as long as wide, pronotum 1.05 times as long as wide, elytra 1.1 times as long as wide, 1.1 times as long as pronotum; body reddish-brown, faintly shining, covered with pale hairs. In general similar to male, except front much more strongly convex and bearing a brush of middle-sized brownish hairs set in form of horseshoe. Upper margin of this brush attains upper level of eyes. Above upper portion of hair-brush overhangs one more brush of brown hairs originated from vertex. As in male puncturation of second and third abdominal sternites is uneven, punctures on these sternites located only on separate slightly deepened areas separated by dull slightly elevated surface without puncturation. Surface of second and third sternites dull, fourth glossy and shining. Second abdominal sternite is covered by densely set yellow hairs of moderate length; lateral sides of third sternite are armed with blunt tubercles. Fifth sternite medially impressed.

#### Notes.

There are two males of Scolytus amazonicus preserved in the collection of K. Schedl. The specimen labeled as a female has a front with damaged frontal vestiture. Due to the front structure with the short brush of hairs, this specimen was erroneously treated as female. Based on the female trapped in Peru, both sexes are distinguished by the features described above. In S. L. Wood’s monograph (2007), descriptions of both male and female are incorrect.

### Scolytus angustatus

Browne, 1970

Figs 3 4

Scolytus facialis Schedl, 1973, syn. n.

#### Material examined.

Brazil: Santarem, Holotype of Scolytus angustatus Browne ♂, BMNH; Maturaca, Amazonas, alto Rio Cauaburi, 12–17.XII.1962, J.Bechyne, Holotype of Scolytus facialis Schedl ♀, NHMW. Peru: Loreto province, 60 km SW from Iquitos to Nauta, Itaya river, 120 m, S4°11'; W73°26', 9–12.02.2007 A. Petrov (2♂♂, 2♀♀)

#### Diagnosis.

The male of species is related to male Scolytus costellatus Chapuis, distinguished by small punctures of frons, by the shorter, transverse costa in the basal centre of second abdominal sternite, in male lateral parts of second and third sternites with sharpened tubercles; in female front with median elevated line, longitudinal impression in upper portion of front and with two orange fringes overhanging front from vertex; front of female with slightly elevated median line from epistoma up to center of front, vertex with two symmetrically orange fringes

**Figure 3.**
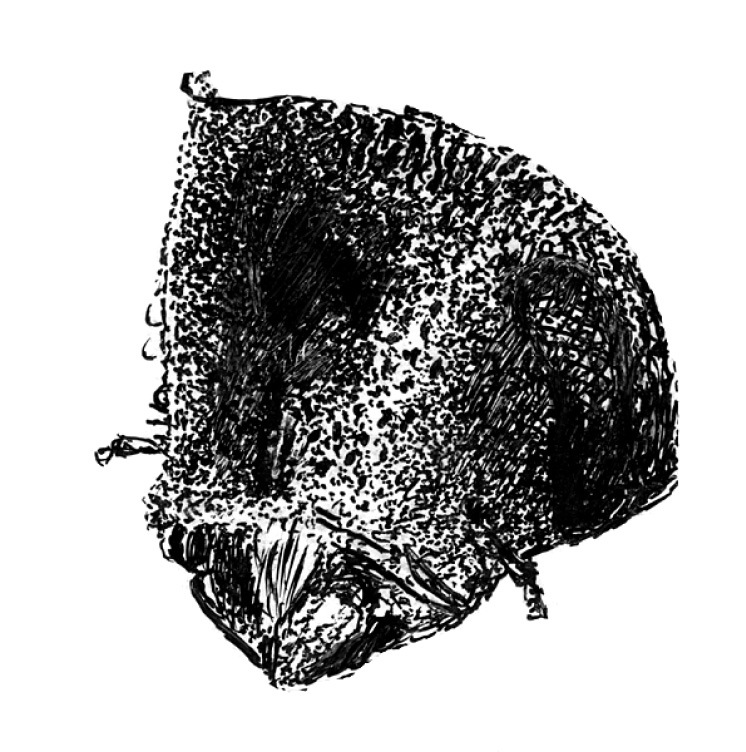
Head of Scolytus angustatus male

**Figure 4.**
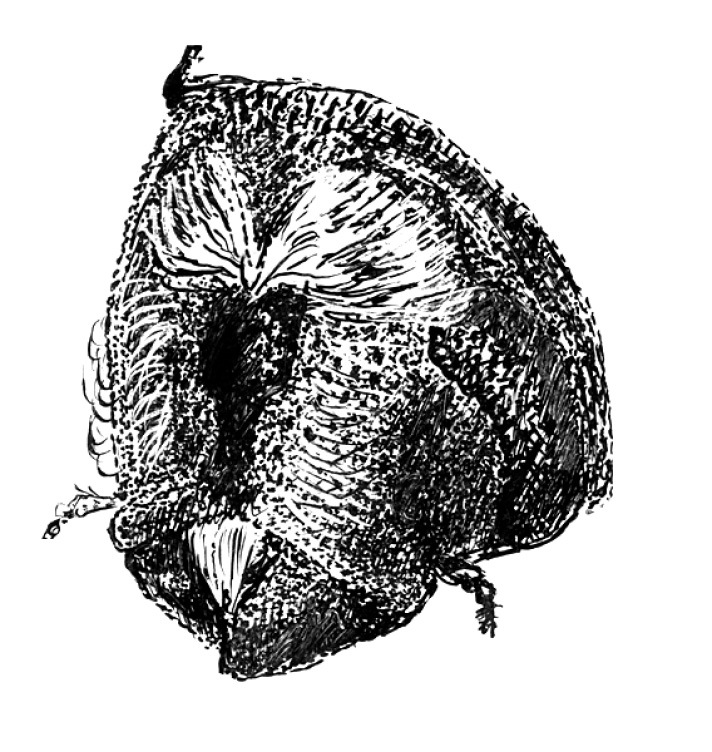
Head of Scolytus angustatus female

#### Description.

##### Male:

body length 2.9–3.6 mm, 2.1–2.2 times as long as wide; body reddish-brown, shining, covered with pale hairs. Head reddish brown with black mandibles and brown antennae. Front slightly convex, nearly flat, upper half feebly flattened, lower half with a weak median crest. Front minutely punctured, more abundant at lateral parts of front and above mandibles. Lateral sides of front evenly rounded and covered by long yellow recumbent hairs forming a brush. At upper part of front, hairs sparser. Central frontal area nearly devoid of hairs, its lower part with small keel-like tubercle. Antennae brown, club of elliptical form, longitudinally elongated. Pronotal length is approximately equal to its width, 1.0–1.1 times as long as wide, reddish brown, its surface shining, evenly punctured; in apical portion of pronotum points are larger and set denser. Apical portion of pronotum lateral sides with recumbent pale hairs. Pronotum separated from prothorax by a well-defined edge. Puncturation of lateral surface of prothorax (propleura) is shallow, unconspicuous. Prothorax covered by sparse, recumbent pale hairs.

Scutellum of triangular form, set deeply in scutellar depression, covered by short pale hairs.

Elytra 1.0–1.1 times as long as wide, 1.0–1.2 times as long as pronotum; striae distinctly, narrowly impressed; interstriae about three times as wide as striae. Interstrial punctures are small, organized into regular rows; there are larger fovea between the small punctures of interstriae; fovea with short pale hairs. Vestiture of elytra at interstriae of rather stout hairs, some rows extending to basal half (2, 5, 7), in other hairs present only near declivity (1, 3, 4, 6).

Central portion of first abdominal sternite curved backwards forming an arc. Second sternite is considerably impressed in relation to posterior margin of first sternite. Lateral sides of second sternite posterior margin with small denticles. Third, fourth and fifth abdominal sternites form an angle of 70º with first sternite. Lateral sides of third and fourth abdominal sternites carry pair of blunt small tubercles each. Fifth sternite is broadly impressed, with sharply elevated rim at posterior margin. Sternite surface is dull, faintly shagreen, covered with erect golden hairs of moderate length. Fifth sternite glabrous. Legs are reddish-brown, covered by short pale hairs.

Female differs from male by frontal structure and vestiture and also by form of abdomen.

##### Female:

frons convex, without median tubercle, median one-fourth moderately sulcate from near middle of frons to vertex; lateral sides of female front are covered by densely set brown hairs of moderate length. Upper margin of frontal brush attains the upper margin of eyes; here paired bundles of longer, densely set hairs from vertex overhang frontal brush; apices of these bundles directed one to another. Elytra are essentially as in male, striae narrowly impressed; interstriae about three times as wide as striae. Vestiture of elytra in some rows extending to basal half (2, 5, 7), in other only near declivity (1, 3, 4, 6). Posterior margin of first segment is thickened and curved backwards in the form of horseshoe; second abdominal sternite set vertically. Lateral sides of abdominal sternites unarmed. Sternites are covered with long pale hairs with their apices curved.

##### Notes.

S. L.Wood (2007) treated Scolytus facialis as a separate species but did not include it into his species key due to the fact that “the exact placement of this species in classification cannot be determined until the male is found” (2007). Findings in Peru have provided males and solved the question of the placement of Scolytus facialis in the genus.

### Scolytus antennatus

Schedl, 1935

Fig. 5 

#### Material examined.

Brazil: Sao Paulo. Bahia, Cepec, Ilheus, 1.III.1961, blacklight, Kaston; Mato Grosso, Xingu, XI-1961, Alvarenga & Bokerman NHMW; Peru: Loreto province, 20 km NE from Iquitos, Momon river, Gen Gen vill.,120 m a.s.l., 6.02.2007. A.Petrov (1♂, 1♀).

**Figure 5.**
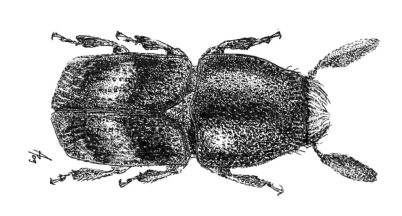
Habitus of Scolytus antennatus

#### Diagnosis.

This species differs from other species of Scolytus by the strongly enlarged and elongated club of antennae in both sexes; club is more than three times longer than the combined length of scapus and funiculus; also by the oblique light band on the elytra and by the pecularitites of abdomen in male and in female.

#### Description.

##### Male:

Length 1.9 –3.1 mm, 2.1–2.6 times as long as wide; color reddish-brown. Front broadly flattened from epistoma to well above upper level of eyes; surface finely aciculate; all frontal surface is covered by pale hairs, at sides of front and in its upper part hairs are thicker and longer, their apices bend towards frontal centre; epistoma with brush of hairs overhanging mandibles. Antennae reddish-brown with strongly elongated club, length of club 0.7 mm, club 2.3 times as long as wide; length of club 3 times greater than the combined length of scapus and funiculus. Pronotum reddish-brown, 1.0–1.1 times as long as wide; surface shining, punctures small on base and disk, 3 times as large near anterior margin, larger in lateral areas of pronotum; anterior margin with sparse pale hairs. Lateral sides of the prosternite (propleura) abundantly punctured with small points bearing short, pale, scale-like hairs, punctures of prosternite are significantly smaller compared to punctures of lateral portion of pronotum.

Elytra bicolorous, central portion of elytra with an oblique light band. Elytra 1.2 times as long as wide; 1.1–1.3 times as long as pronotum; elytral puncturation is very variable, diameter of punctures may differ by 1/3 of diameter in separate specimens. Punctures form linear striae, the striae feebly to not impressed; interstriae smooth, shining, punctures very small. Declivity very weak with sparse, short, pale recumbent hairs. Abdomen is reddish-brown. Second abdominal sternite oblique, angle with first sternite obtuse, anterior margin of second sternite weakly elevated, subcostate, fifth sternite elevated in anterior portion, with a large, weakly compressed median spine on anterior half, in posterior portion fifth sternite is slightly impressed. Abdominal surface dull, very faintly shagreen, pale hairs at abdomen sparse and short, apices directed towards median portion of sternites. Legs reddish-brown with sparse hairs.

##### Female:

similar to male except frons convex on upper half, vestiture less abundant, but much longer on upper half; second sternite with large dark tubercle of triangular form occupying median portion of sternite from its anterior margin up to the middle; fifth sternite unarmed.

#### Notes.

The species is recorded from Peru for the first time. The specimens were collected in the barrier trap set at a forest clearing.

### Scolytus bicinctus

Schedl, 1972

Fig. 6 

#### Material examined.

Brazil:Jacareacanda, Para, VI-1970, ER Barbosa. M.Alvarenga Collection.Holotype ♂; Peru: Loreto province, Morannon river, 20 km NNW from Nauta, Buen Fin vill., 130 m, 6.02.1995 A.Petrov (1♀); Junin province, Rio Perene, 8 km NNE from Puerto Ocopa, Cananeden vill., 1180 m, S11°49'; W74°16' 6.02.2008 A.Petrov (1♂).

**Figure 6.**
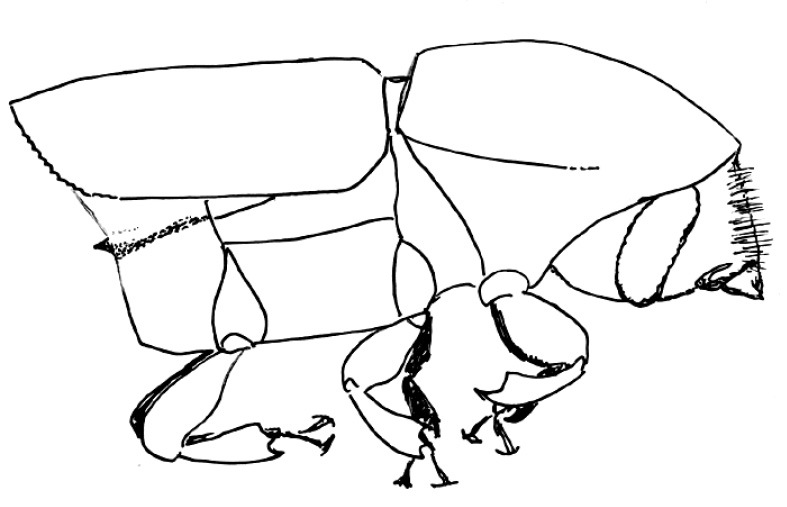
Habitus, lateral view of Scolytus bicinctus, female

#### Diagnosis.

Species differs from its relatives by structure of abdomen, by frontal and elytral puncturation.

#### Description.

##### Male:

body length 1.5–1.9 mm, 2.3 times as long as wide; body colour from reddish-brown to grayish-brown; surface faintly shining, nearly dull. Head reddish-brown or grayish-brown. Front broadly, rather strongly convex, surface obscurely reticulate, moderately punctured over entire area; vestiture of sparse, fine, short hairs, with most hairs located on lateral frontal parts and above mandibles; scape and funicle are reddish brown, lighter compared to club, antennal club with acutely angulate groove for suture 1 clearly marked, apex of club is evenly rounded. Pronotum 1.04–1.1 times as long as wide, its lateral sides parallel from basis up to middle of its length, towards apex lateral sides are strongly narrowed; pronotal surface faintly shining, evenly punctured by shallow punctures; these punctures becoming larger in apical portion of pronotum; sparse pale hairs located in apical portion of pronotum. Pronotum is separated from the prosternite (propleura) by a poorly developed, obtuse and smooth margin. Puncturation of lateral sides of prosternite very shallow and unconspicuous. Prosternite covered by small recumbent scale-like, pale hairs.

Scutelum triangular, set not deep in scutellular impression below elytral surface.

Elytra 1.3–1.4 times as long as wide, 1.25–1.3 times as long as pronotum; lateral sides of elytra nearly parallel up to short declivity, from beginning of declivity and up to suture elytra are narrowed, with their sides forming a 45° angle. Striae and interstriae weakly impressed, punctures in striae and interstriae about equal in size. Elytral surface from basis and to the apex is evenly covered by very short pale recumbent hairs. Central portion of posterior margin on first sternite and second sternite basis faintly projected backwards; the border between first and second sternites unevident. Basis of the second sternite with two unconspicuous callus-like tubercles, lateral sides of the first sternite are narrowed. Lateral denticles of the second sternite posterior margin with clearly attenuated apices, conspicuous; second sternite is nearly vertical towards posterior margin of the first sternite. Third, fourth and fifth sternites forming angle of 45° with first sternite. Lateral sides of third and fourth sternites unarmed. Fifth sternite impressed near its posterior margin, the posterior margin forming an elevated rim. Surface of sternites is faintly shining, finely shagreen (reticulated) and evenly covered by recumbent pale hairs.

Legs are reddish brown, covered by sparse short hairs.

Female differs from male by more cylindrical form of body, by structure of front and of abdomen.

##### Female:

body length 1.8 mm, 2.5 times as long as wide; color reddish brown. Front convex, without tubercles or impressions, its surface finely shagreen and evenly punctured, in central portion front has light transverse wrinkles; all frontal surface in female covered by short pale hairs; longer singular hairs on epistoma overhanging the mandibles. Apical constriction of the pronotum poorly developed; therefore pronotum seems to be broader than in male. Elytra essentially as in male, their surface is covered with minute pale recumbent hairs. Central portion of the first abdominal sternite is weakly projected backwards, its posterior margin smooth. Lateral sides of first sternite narrowed, second sternite much broader compared to the first. Second sternite set nearly vertical towards posterior margin of first sternite. Lateral portions of posterior margin on second sternite with small horizontal sharpened denticles. Third, fourth and fifth sternites forming a 70° angle with first sternite. Lateral sides of third and fourth sternites unarmed. Fifth sternite with median impression nearb posterior margin, and margin with an elevated keel. Surface of abdominal sternites faintly shining, finely shagreen and evenly covered by short recumbent pale hairs.

#### Notes.

The species is found in Peru for the first time. Female is described for the first time here.

### Scolytus canellae

Wood, 2007

Fig. 7 

#### Material examined.

PERU: Loreto province, left bank of Amazon River 58 km SW from Iquitos to Nauta, Itaya river, 120 m a.s.l., 9.V.2009. A.V. Petrov (3 ♂♂).

**Figure 7.**
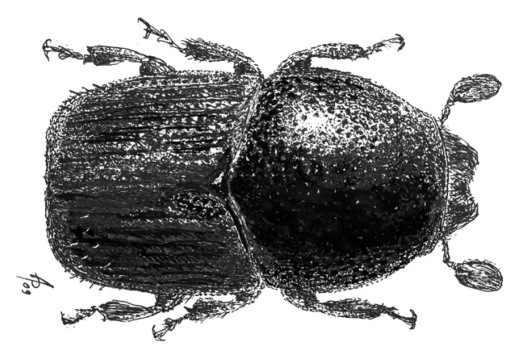
Habitus of Scolytus canellae ,male

#### Diagnosis.

The species can be distinguished from other representatives of the genus by body shape, and by the form and position of the tubercle on the second abdominal sternite.

##### Male:

body length 3.0–3.3 mm, 1.7–1.9 times as long as wide; body black, shining. Head black, its surface shining. Front transversely flattened eye to eye, longitudinally weakly convex from epistoma to vertex. Frontal surface densely longitudinally aciculate and with small shallow punctures between wrinkles. Median line slightly below frontal center weakly elevated, forming an inconspicuous tubercle (in some specimens with faintly elevated longitudinal keel). Frontal surface with densely set brown hairs, at lateral sides of front these hairs longer, their apices oriented towards center of the front. Median line devoid of hairs. Antennae reddish-brown or grayish-brown. Club of elliptical form with evenly rounded apex, covered with densely set short yellowish hairs. Pronotum 0.85–0.9 times as long as wide. Its surface smooth, shining, evenly punctured on base and on disc, punctures being larger near anterolateral angles. Pronotum is divided from propleura by the well developed acute margin. Lateral sides of prothorax (propleura) densely and evenly punctured by punctures equal to those at lateral margin of pronotum. Pronotum and propleura glabrous, with only 2–3 hairs at anterolateral margins.

Scutellum of triangular form, set deeply in scutellar impression.

Elytra black, 0.65–0.9 times as long as wide, 0.9 times as long as pronotum, striae and interstriae narrowly, about equally impressed, interstriae punctures spaced by 2 diameters of a puncture, punctures separated by diameter of puncture. Posterior elytral margin with sparse, short erect hairs forming short rows. Abdomen black, its surface dull, all sternites are evenly punctured, punctures on second sternite are slightly larger than punctures at sternites 3–5; width of second sternite two times greater than length, second sternite vertical in relation to first sternite, with a very small, median spine near posterior margin; fifth sternite with elevated posterior margin; sternites are covered with short erect brown hairs. Legs reddish-brown with short pale hairs.

##### Female:

Similar to male except front more finely sculptured; second sternite without spine (Wood, 2007).

#### Notes.

We have not seen female specimens. S.L. Wood described the species from pale teneral beetles, the mature imago is black.

### Scolytus carveli sp. n.

urn:lsid:zoobank.org:act:B05AF2DF-0F7F-4378-81DF-F9545C426469

Figs 8 12 

#### Type locality.

Peru, Loreto province, left bank of Amazon River, Itaya River, S 04.15.510 W 073.28.032.

#### Type material.

Holotype ♂ (ZMM): PERU: LORETO PROVINCE: Itaya river, left bank of Amazon River, 58 km SSW from Iquitos to Nauta, 120 m a.s.l., 73°26'W; 4°11'S, 17.02.2008, leg.. A.Petrov. **Paratypes**: 1♂, 1♀ (Petrov collection): PERU: LORETO PROVINCE: Itaya river, left bank of Amazon River, 58 km SSW from Iquitos to Nauta, 120 m a.s.l., 73°26'W; 4°11'S, 17.02.2008, leg.. A. Petrov (1♂); PERU: LORETO PROVINCE: 70 km SW from Iquitos to Nauta, 130 m a.s.l., 27.02.2008, leg.. A. Petrov (1♀).

**Figure 8.**
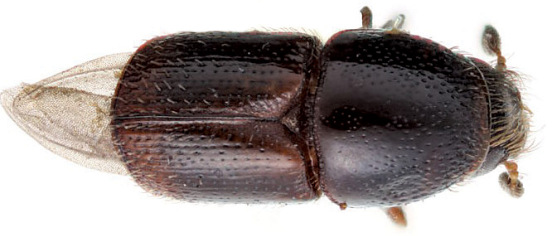
Habitus of Scolytus carveli, male

**Figure 9.**
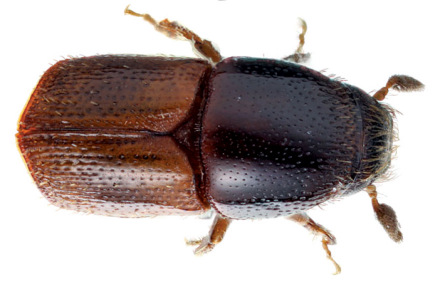
Habitus of Scolytus carveli, female

**Figure 10.**
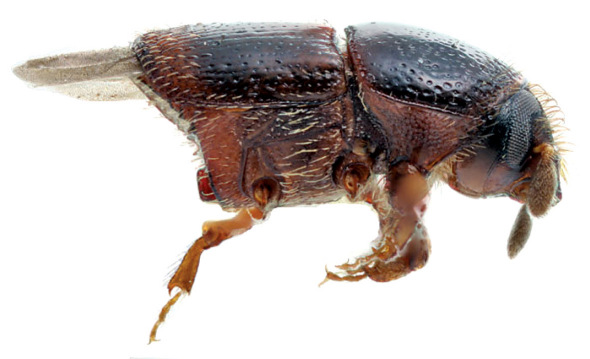
Habitus, lateral view of Scolytus carveli, male

#### Diagnosis.

This species is morphologically closely related to Scolytus vagabundus sp. n., from which can be distinguished by body size, by shape of carina-like tubercle and its position on second sternite; by absence of a median tubercle on fifth sternite and by abdomen vestiture character. The species differs from Scolytus pinnatus by its size, by absence of tubercles and denticles on fourth sternite and by other features described below.

#### Description.

##### Male:

body length 1.9 mm, 2.3 times as long as wide; color dark brown, elytra reddish brown. Front faintly convex in central portion and faintly impressed in epistomal area; frontal surface faintly shining, longitudinally aciculate. Lateral sides of front covered by sparse grey hairs of moderate length, the hair apices directed towards frontal center. Antennae brown, covered by short golden hairs, club of elliptic form with the evenly rounded apex. Pronotum of equal length and width. Pronotum dark brown in colour, its basis more light in colour, reddish-brown; pronotal surface shining, punctured by small shallow punctures at basis and in central portion of disk; punctures on apical portion of pronotum and its lateral sides large and deep. Pronotum is separated from prosternite by the well-developed sharp lateral margin. Lateral sides of prosternite (propleura) punctured densely and evenly by punctures of moderately large size. Sparse, erect hairs limited to apical portion of pronotum.

Scutellum triangular, deeply set in the scutellar impression.

Elytra reddish-brown, central part darker, which gives an impression of a banded elytra. Elytra 1.28 times as long as wide, 1.0 times as long as pronotum; lateral sides of elytra nearly parallel up to short declivity, from beginning of declivity and up to sutural apex elytra sides narrowed forming an angle of 45°. Elytral suture slightly elevated from scutellar impression and up to elytral apex. Elytral surface shining, punctured with regular rows of punctures, in posterior third of elytra striae slightly impressed. Interstriae flat and smooth, with sparse punctures, conspicuous only in posterior part of elytra. Posterior third of elytral interstriae with rows of pale, erect hairs. Abdomen reddish-brown. Second sternite set vertical, perpendicular to first sternite, anterior margin of second sternite weakly elevated, costate, second sternite armed by large, laterally compressed median carina, its apex truncate. Carina occupies position from basis of second sternite and up to its centre. Lateral sides of second and third sternites with small denticles, denticles on third sternite smaller and with a blunt apex. Surface of abdomen shining, punctured with very small punctures, covered with sparse short erect hairs. Legs reddish brown, covered by golden hairs.

##### Female:

similar to male except front more convex, vestiture less abundant and shorter, abdominal sternite without carina, lateral sides of second and third sternites with small denticles as in male, on second sternite denticles with sharp apex, on third sternite denticles smaller and with blunted end.

**Figure 11.**
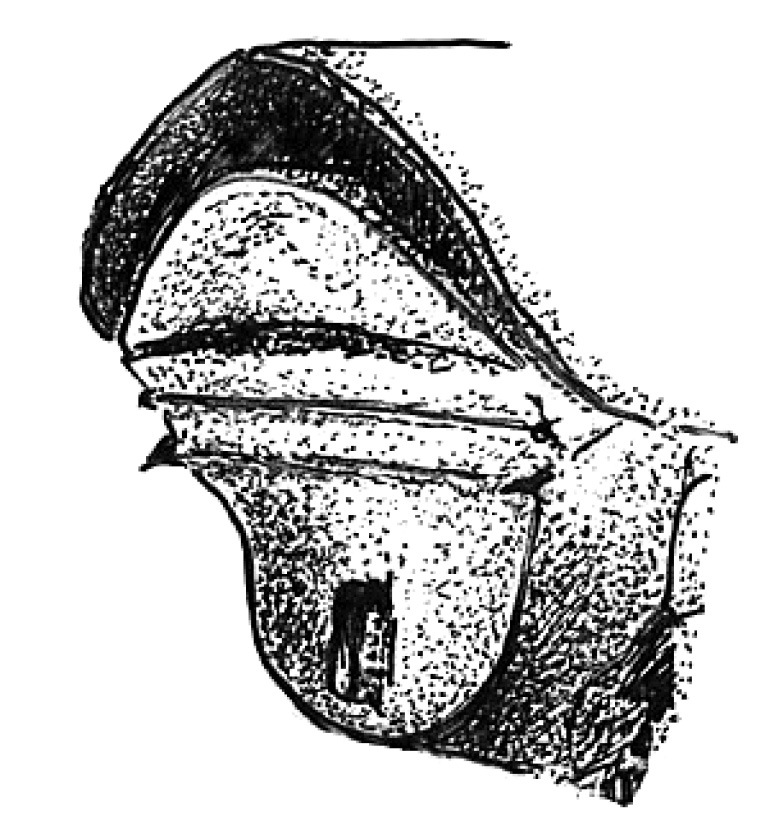
Sternites of abdomen of Scolytus carveli, male

**Figure 12.**
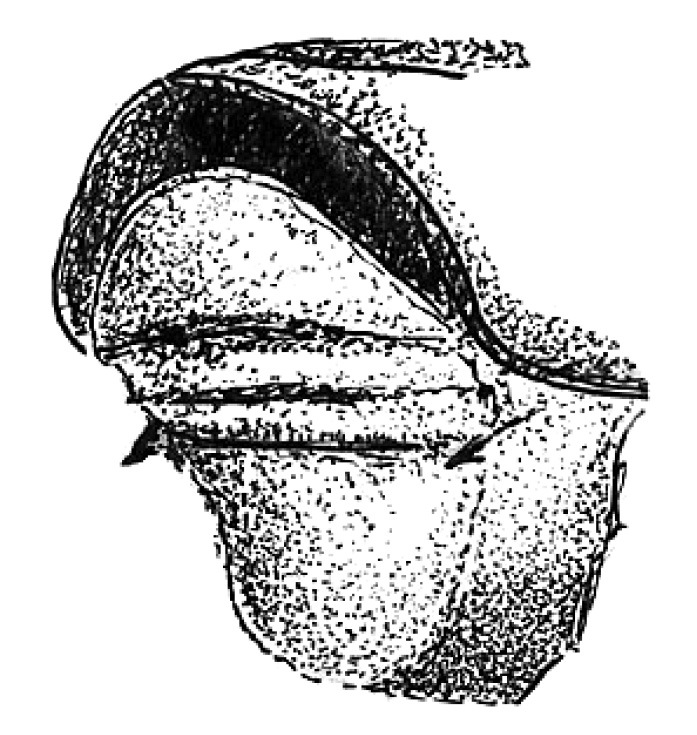
Sternites of abdomen of Scolytus carveli, female

#### Notes.

Males have been collected into barrier traps set on Protium, that were cut three days before beetle collecting.

#### Distribution.

Known only from the type locality.

#### Etymology.

The species name relates to the similarity of the body of the bark beetle to the old ship carvel.

### Scolytus costellatus

Chapuis, 1869

Fig. 13 

Material examined: Peru: Loreto province, 58 km SW from Iquitos to Nauta, Rio Perene, 120 m a.s.l., 11.02.2005 A.Petrov (3♀♀), same locality, but 9–12.02 2007 (3♂♂, 2♀♀), same location but 5–8.02.2008 A.Petrov (1♂♂, 4♀♀). Junin province, Perene river, 11 km from Puerto Ocopa vill, Los Olivos, 1180 m a.s.l., 11°3.00'S; 74°15.52'W 26–31.03.2009 A.Petrov (1♂, 2♀♀). Cusco province, 4 km SW from Machu Picchu, 1300 m, 21.IV.2009, A.Petrov, (1♀)

**Figure 13.**
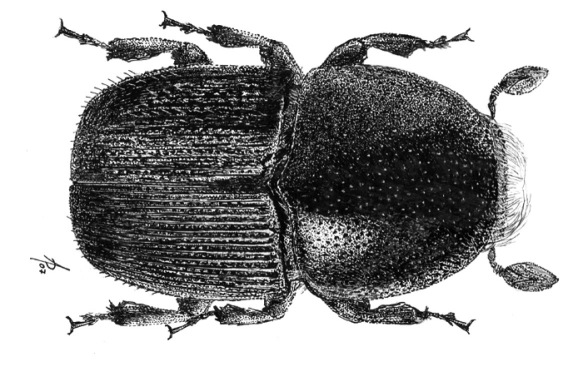
Habitus of Scolytus costellatus, male

#### Diagnosis.

Species differ from all other representatives of genus by the narrowed, pointed antennal club and by the reduced scutellum. Also diagnostic for the species are equally deepened elytral striae and interstriae, male second sternite with nearly sharp transverse carina, occupying most portion of second sternite base.

#### Description.

##### Male:

body length 3.2–4.1 mm, 1.8–1.86 times as long as wide; body black, shining. Head black, faintly shining, with dark brown mandibles. Front flat, evenly longitudinally aciculate from vertex and to lower portion of epistoma. Central portion of front is covered by sparse golden hairs, at lateral sides and on upper margin of front these hairs thicker and longer, with their apices directed towards the centre of front, forming a golden brush. Antennae with reddish-brown scapus and two first funicular segments; club and segments 3–7 of funiculus dark grayish-brown. Club with a narrowed and pointed apex covered with short gray hairs (Fig. 13). Pronotum 0.9–1.0 times as long as wide, central part of basis with the projection overhanging above scutellum, its surface smooth and shining, with small punctures at base and in central part, at anterolateral angles, punctures are larger and of moderate size. Apical margin of pronotum with sparse thin and short hairs. Pronotum is divided from prosternite by the well-developed acute lateral margin. Lateral sides of pronotum (propleura) are abundantly and evenly punctured with punctures of size equal to size of punctures at lateral margins of pronotum.

Scutellum is reduced, nearly obsolete.

Elytra 0.9–1.0 times as long as wide, 0.9–1.1 times as long as pronotum, striae and interstriae are equally sulcate from base to declivity of elytra; punctures in striae and interstriae are small, about equal in size, spaced by diameter of a puncture; entire elytral surface covered by short, erect, dark setae. Declivity weak, with strongly confused puncturation. Abdomen black, its surface nearly dull, sternites abundantly and densely punctured by punctures of different size, punctures on second sternite are four-time larger than punctures on sternites 3–5. Transversal length of second sternite is two-times greater its longitude, second sternite set subvertical to first sternite, its anterior margin subacutely costate on median area; fifth sternite with weakly elevated posterior margin; sternites covered by erect pale moderately long hairs. Legs black, tarsi reddish-brown, meso- and metafemora with long pale hairs.

##### Female:

similar to male except front weakly convex, frontal width and form are very variable, in some females in upper portion of front there is a thin median line, vestiture in lateral margins shorter but abundant; second sternite with costa absent, its base rounded, anterior margin with small bifurcated callus, erect abdominal setae shorter than in male.

#### Notes:

After examination of the type specimens of Scolytus pseudocostellatus and Scolytus strigipennis we concluded that both species are junior synonyms of Scolytus costellatus. The features considered as species-specific fall into intraspecific variability of Scolytus costellatus.

#### Host.

Liana.

#### Biology.

Infest the lianas that were mechanically damaged. The egg galleries very long, longitudinal, biramous. Egg chambers are located strictly on one side of the egg gallery. Number of egg chambers varies from 55 to 95. The larval galleries are perpendicular to the main egg gallery, do not cross one another, form a circle around the liana stem and run back towards the egg gallery from another side where deepen into xylem, where the pupal chambers are formed.

### Scolytus cristatus

Wood, 1969

#### Material examined.

Peru: Junin province, Perene river, 8 km from Puerto Ocopa vill, 1180m a.s.l., Cananeden vill. 1180m a.s.l., 11°4.92'S; 74°16.10'W, 2–7.02.2008, A.Petrov, (11♂♂7♀♀), same locality 5.IV.2008 A.Petrov (4♂♂4♀). Junin province, Perene river, 11km from Puerto Ocopa vill, Los Olivos, 1180m a.s.l., 11°3.00'S; 74°15.52'W 25–26.03.2009 A.Petrov (1♂2♀).

#### Diagnosis.

Second abdominal sternite is armed in males by the strong laterally compressed spine that occupies the upper portion of the segment, but that is touching neither anterior nor posterior margins of the sternite; short bristles simple, hair-like; male front vestiture developed mainly below upper margin of eyes.

#### Description.

##### Male:

body length 2.3–3.3 mm, 2.0 times as long as wide; color dark reddish brown. Front dark brown with shining surface. Front with weak transverse impression above epistoma, flat from there to upper level of eyes; surface aciculate from vertex to epistoma. Median line slightly below lower centre of front weakly elevated. Vestiture of abundant, long hair uniformly distributed, longer nearby frontal margins, median line without hairs. Antennal funiculus and scapus reddish brown. Club brown, covered with densely set golden hairs. Pronotum 1.0 times as long as wide, surface smooth, shining, punctures small, deep on base and disk, much larger on lateral margins of the apical area. Punctures at anterior margin of pronotum not confluent. Surface of pronotum glabrous, devoid of hairs. Prosternite separated from pronotum by well-developed acute lateral margin. Lateral sides of the prosternite (propleura) abundantly and evenly punctured by the punctures of equal size with the punctures at lateral margins of pronotum.

Scutellum triangular, set not deeply into scutellar impression.

Elytra 1.0–1.1 times as long as wide, 1.1 times as long as pronotum; striae impressed, first and second interstriae impressed from base of elytra, all other interstriae are impressed from basal third or from more distal part of elytra; punctures in striae and interstriae small, about equal in size, spaced by diameter of a puncture. Posterior elytral margin with unconspicuous rows of short erect hairs. Abdomen reddish-brown, its surface dull; second sternite subvertical, with a black median, laterally compressed carina, occupying central half of segment, carinate tubercle is evenly rounded at base, its highest point near posterior margin. Surface of second abdominal sternite with evenly placed circular punctures, punctures of other sternites lesser in size compared to punctures of second sternite; complete abdominal surface evenly covered by short erect hairs. Legs reddish-brown, covered by recumbent yellow hairs.

##### Female:

similar to male except front much more strongly convex; frontal hairs are short and sparse, conspicuous only at epistoma; carina on second sternite smaller.

#### Host.

liana

#### Biology.

The species infests lianas that were mechanically damaged. Parental tunnels are biramous and transverse; the larval mines are longitudinal. Number of egg chambers varies from 15 to 45. Larval galleries are longitudinal, long. Complete cycle of development from the egg to teneral adults takes 45 – 56 days.

### Scolytus lindemani sp. n.

urn:lsid:zoobank.org:act:1AF1664C-62A1-45F9-A028-B4CA62AF9AD7

Figs 14 15 

#### Type locality.

Peru, Loreto province, left bank of Amazon River, Itaya River S04.15.503 W073.28.035, 62 km SW from Iquitos.

#### Type material.

Holotype ♀ (ZMM): PERU: LORETO PROVINCE: Itaya river, left bank of Amazon River, 58 km SSW from Iquitos to Nauta, 120 m a.s.l. 3.02.2006 leg.. A.V. Petrov.

**Figure 14.**
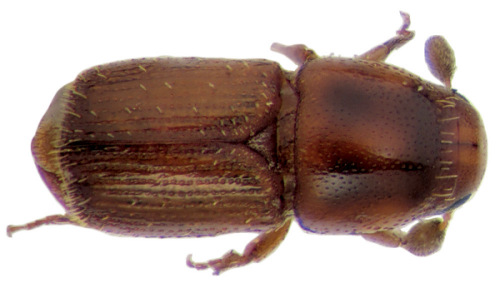
Habitus, dorsal view of Scolytus lindemani, female

#### Diagnosis.

Differs from Scolytus spinidens by the shape of the abdomen and by the armature of the second abdominal sternite.

#### Description.

##### Female:

body length 2.0 mm, 2.5 times as long as wide; color reddish brown. Front light brown, faintly shining, strongly convex from epistoma to vertex. Frontal surface evenly punctured by shallow sparse punctures from epistoma to vertex; vestiture very scanty, represented by sparse short pale hairs. Vertex with narrow dark median line. Antennae brown, club elliptical, covered by short golden hairs. Pronotum 1.0 times as long as wide. Maximal width of pronotum around midpoint, lateral sides parallel through most of their length, at apex with an evident constriction; surface is gently reticulated , faintly shining, at base and on disk with small shallow punctures, punctures larger but also shallow near sides of pronotum and in its apical portion. Pronotum is divided from propleura by the poorly developed elevated margin. Propleura smooth, non punctured. Very sparse hairs are concentrated in apical portion of pronotum.

Scutellum triangular, scutellar impression is not developed.

Elytra light brown in color, 1.3 times as long as wide, 1.3 times as long as pronotum; lateral sides of elytra nearly parallel up to posterior part, where rather suddenly narrowed towards sutural apex, with their sides forming an angle of 45°. Elytral suture is slightly raised. Elytral surface dull, covered with regular rows of punctures of medium size. Interstriae flat and smooth; second, fourth, sixth and eighth interstriae with very sparse punctures set far apart from the neighbours; first, third, fifth and seventh interstriae without puncturation. Sparse punctures of the interstriae carry one short scale-like pale hair each. Near elytral apex, hairs longer and more abundant. Abdomen light brown in colour. Second sternite set vertical, perpendicular to first sternite, junction between first and second abdominal sternites rounded, with no indication of a transverse carina, suture between first and second sternites poorly marked, blurred, practically invisible. Second sternite armed by 5 sharpened denticles: near the posterior margin, a conical, pointed median spine is located, which does not touch the posterior margin of the sternite; lateral sides of second sternite with two sharp denticles; at lateral sides of the border between first and second sternites there are two additional sharp tubercles, these tubercles separated by 2/3 of sternite length; the rest of sternites unarmed. Central portion of fifth sternites impressed, its posterior margin raised. Surface of sternites roughly punctured by multiple punctures. Abdomen covered by short pale scale-like hairs. Legs brown, covered with pale hairs.

##### Male:

Unknown

**Figure 15.**
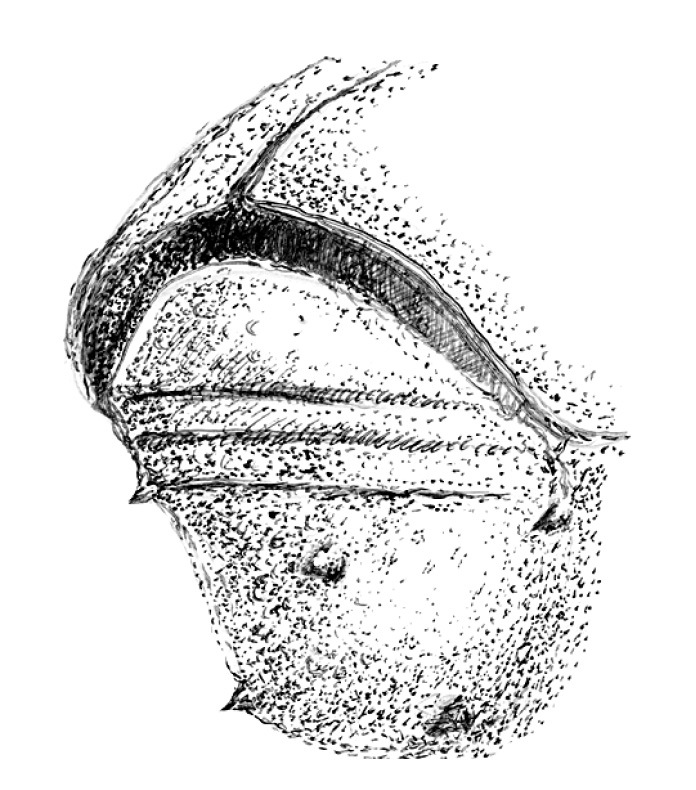
Sternites of abdomen of Scolytus lindemani, female

#### Distribution.

known only from the type locality.

#### Etymology.

The new species is named in honor of the Russian forest entomologist G.V. Lindeman, who dedicated his life to studies of xylobiont insects.

### Scolytus excavatus

Wood, 2007

Description is based on specimens collected in Peru and provided by Sarah Maria Smith.

Peru Madre de Dios, Los Amigos Biological Station, GPS: S12°34'9"; W70°6'0.40", 19–25.V.2008 Smith & Hulcr Colls.

#### Description.

##### Male:

body length 5.5–6.4 mm, 1.8–2.0 times as long as wide; color red brown to black. Front concave eye to eye from epistoma to well above upper level to eyes, mandibles at base are strongly thickened and elevated, with blunt tubercles, with deep transverse furrows and rugositites, frontal margin above mandibles strongly incurved towards central portion of the front, center of front with small median carinate tubercle, front above this tubercle with the circular impression that occupies space up to upper level of eyes, bottom of the impression with the surface smooth, finely punctured. Sides of impression are covered with yellow erect bristles not forming dense brush, in the upper portion of front hair-like vestiture is sparse, in several specimens represented by singular hairs only; antennae brown, club elliptical with evenly rounded apex, covered by short golden hairs. Pronotum 0.8–0.95 times as long as wide, its base with the median projection towards scutellum, lateral sides of prontoum are evenly narrowed from base to apical margin, without subapical constriction; pronotal surface faintly shining, nearly dull, covered by minute punctures, diameter of punctures at the sides of pronotum slightly larger that diameter of punctures in its centre, apical margin of pronotum with sparse hairs.

Scutellum very small, triangular, immersed deeply into scutellar impression.

Elytra 0.8–1.0 times as long as wide; striae impressed, strial punctures of moderate size, not confluent. Interstriae about tree times as wide as striae, covered by small punctures with few scattered setiferous pores bearing one erect thick short bristle each. Posterior elytral margin with only sparse puncturation. Abdomen black, its surface dull, evenly punctured by punctures of moderate size, vestiture abundant, entire surfaces of abdominal sternites 2–4 covered by long yellow hairs, surface of the fifth sternite with sparse hair-like vestiture except on lateral sides of the fifth sternite base. Border between first and second sternites junction strongly projected backwards and also elevated, shape of the second sternite is cup-formed incurved, median part of second sternite anterior margin with the shark back fin-shaped denticle curved upwards; this denticle occupies the spase from base of sternite and up to center of the sternite. Fifth abdominal sternum is medially impressed, its posterior margin is elevated.

Legs black, tarsi reddish-brown; femora are covered by short recumbent hairs, outer margin of meso- and metatibia with long erect yellow hairs

##### Female:

Body length: 4.8–5.3 mm, 1.7–2.0 times as long as wide. Front concave eye to eye from epistoma to well above upper level to eyes, each mandible at the base bear processes with apices directed upwards and towards the apex of the process of adjoining mandible (in older beetles these processes may be broken off); center of the front with deep longitudinal impression continuing upwards up to upper level of eyes, lateral sides of the impression are densely covered with pale hairs. Lateral margins of front are elevated, curved near eyes, following the upper level of eyes line. Frontal surface smooth, gently punctured, at lateral sides of front pale hairs are set denser. From the vertex two symmetrically set fringes (forelocks) of long dark grayish-black hairs are directed towards center of the front. Pronotum: 0.74–0.96 times as long as wide, essentially as in male. Elytra: 0.8–1.2 times as long as wide and similar to elytra of male. Abdomen is covered by short yellow hairs, second sternite with basal margin rounded, subvertical, middle of segment with a large cylindrical spine. Apex of this spine is thickened and covered by short hairs, fifth sternite concave, its apical margin acute.

#### Host.

Pterocarpus rohrii (Fabaceae).

#### Biology.

bigynous, each gallery is built by 2 – 4 females.

#### Notes.

Male is described here for the first time. S.L. Wood erroneously treated female holotype as a male. Studies of a long series of Scolytus excavatus,collected bySarah M. Smith and Jiri Hulcr, allowed to remove this inaccuracy and to expand the description of the female of this unusual species.

### Scolytus mozolevskae sp. n.

urn:lsid:zoobank.org:act:288F4E7C-A481-42B4-92CD-C450D99BE8A3

Figs 16 18 

#### Type locality.

Peru, Loreto province, left bank of Amazon River, Itaya River, S04.15.503 W073.28.035.

#### Type material.

Holotype ♂ (ZMM): PERU: LORETO PROVINCE: Itaya river, left bank of Amazon River, 58 km SSW from Iquitos to Nauta, 120 m a.s.l., 4°11'S; 73°26'W 8.V.2009, leg.. A.V. Petrov. Paratypes 75♂♂, 51♀♀ (Petrov collection): PERU: LORETO PROVINCE: Itaya river, left bank of Amazon River, 58 km SSW from Iquitos to Nauta, 120 m a.s.l., 73°26'W; 4°11'S, 5–6.02.2005, leg.. A.V. Petrov (7♂♂, 3♀♀); same locality, but 2–10.02.2006, leg.. A.V. Petrov (29♂♂, 44♀♀), same locality, but 28.02.2006, leg.. A.V. Petrov (1♂), same locality, but 10.03.2006 (31♂♂, 1♀), same locality, but 3.02.2007 (4♂♂, 1♀), PERU: LORETO PROVINCE: 70 km SW from Iquitos to Nauta, 130m a.s.l., 23.02.2008 (3♂♂, 1♀); same locality, but 27.02.2008, leg.. A.V. Petrov (1♀).

**Figure 16.**
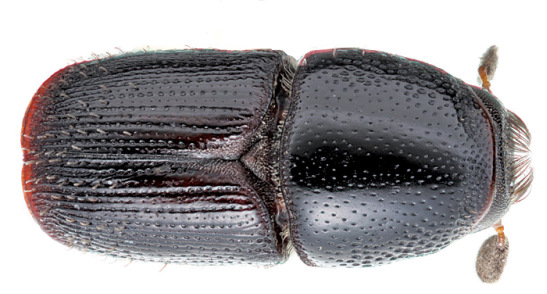
Habitus of Scolytus mozolevskae, male

**Figure 17.**
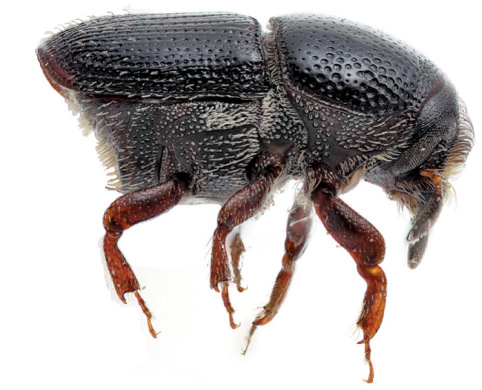
Habitus, lateral view of Scolytus mozolevskae, male

#### Diagnosis.

New species is morphologically closely related to Scolytus amazonicus Schedl from which it differs by body size, by dark hair-like frontal vestiture, by strongly shining second abdominal sternite surface that is punctured by large punctures, by absence of raised callosities and impressed areas with punctures; by absence of lateral tubercles at sides of third abdominal sternite which are smooth.

#### Description.

##### Male:

body length 2.9 mm (paratypes length 2.6 – 3.2 mm), 2.2 times as long as wide; colour dark brown. Front dark, grayish brown with shining surface. Front weakly convex from eye to eye and from epistoma to vertex; surface aciculate from vertex to epistoma. Median line running from epistoma up to upper margin of front well-developed. Frontal surface covered by dense, closely set brown hairs, these hairs darker at base and lighter near apex, hairs above mandibles lighter than hairs at the upper portion of front. Hairs longer at lateral frontal sides, long hair apices directed towards centre of front. Vertex densely punctured by longitudinally elongated punctures. Antennae with reddish brown scapus and funiculus, club with narrow base, gradually widening towards apex, apex of club is evenly rounded. Pronotum black, its surface shining, punctured by sparse deep punctures, at lateral sides punctures larger, closely set compared to punctures of base, disk and apical portions of the pronotum. Apical pronotal area practically devoid of vestiture, with singular short, dark hairs. Pronotum separated from prothorax (propleura) by a clearly marked, sharply elevated pronotal margin. Lateral sides of prothorax (propleura) punctured by points of moderate size, but these punctures smaller compared to those at lateral sides of pronotum. Lateral sides of prothorax are covered by very short scale-like hairs and by sparse setae.

Scutellum triangular, deeply set in scutellar impression, covered by minute scale-like pale hairs.

Elytra dark grayish brown. Elytra 1.15 times as long as wide; 1.24 times as long as pronotum, striae distinctly, narrowly impressed, with round non-confluent punctures, interstriae flat and smooth with rows of small punctures with the diameter significantly smaller than in punctures of rows. At anterior part of elytra punctures are set densely and chaotically. Sparse pale hairs form rows at interstriae of the posterior third of elytral length and throughout complete length at sides of elytra. Abdomen black, its surface shining, uniformly covered by erect moderately long hairs. Second sternite subvertical, nearly perpendicularly set to first sternite, anterior margin slightly elevated, costate. Third, fourth and fifth sternites form a 45° angle with the first sternite. Lateral sides of sternites without tubercles. Second abdominal sternite roughly punctured by deep punctures from its middle and up to the posterior margin; sternite base with sparse punctures only. First, fourth and fifth sternites densely punctured by punctures of middle size. Points at second and third sternites grouped on separately set slightly impressed areas with the elevated surface without any puncturation between. Surface of first and second sternites dull, surface of third and fourth sternites glossy. Fifth sternite slightly impressed medially, its posterior margin elevated. Legs reddish brown, covered by short pale hairs.

##### Female:

body length 2.5–3.1 mm, 2.15–2.2 times as long as wide; colour dark brown. Similar to male except differing in front and abdomen structures. Front broadly convex from eye to eye, from epistoma to vertex; surface aciculate from vertex to epistoma. Vestiture scant, hairs moderately long and sparse, mostly evident at lateral sides of front. Abdomen more convex compared to male abdomen due to slightly rounded first sternite posterior margin and slight decrease in size of second sternite.

**Figure 18.**
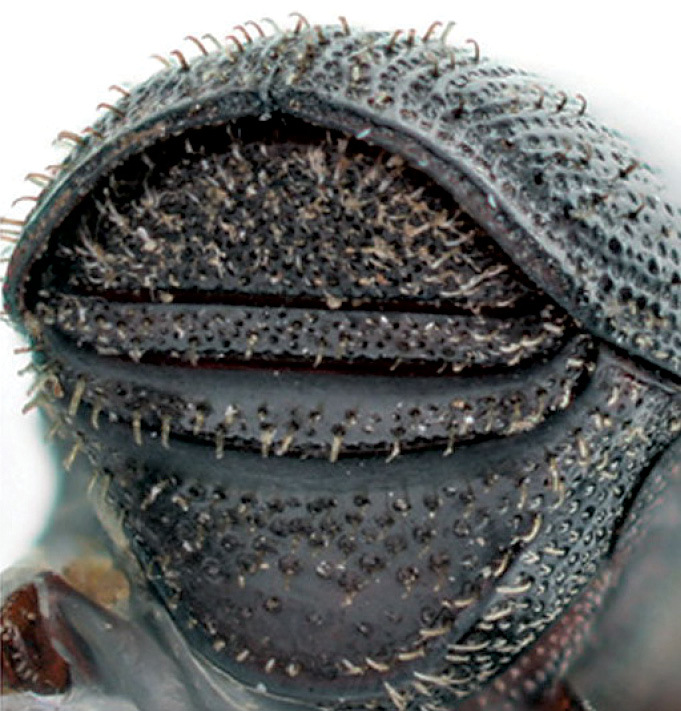
Punctation of second sternit of Scolytus mozolevskae

#### Host.

Liana.

#### Biology.

Scolytus mozolevskyi infests damaged and irreversibly weakened lianas. Beetles infest the liana trunk throughout all its length. Density of galleries is very high, with up to five egg galleries per square decimeter. Parental tunnels are biramous and transverse; larval mines are longitudinal.

#### Distribution.

Known only from the type locality.

#### Etymology.

The new species is named in honor of the forest entomologist Dr. E.G. Mozolevskaya.

### Scolytus neofacialis

Schedl, 1976

Figs 19 20 

#### Material examined.

Brazil: Varginha, M. Gerais, II-1972, M. Alvarenga Holotype ♂, NHMW, Wien. Peru: 20 km NE from Iquitos, Momon river, Gen Gen vill. alt. 120 m, 6.02.2007, A.Petrov (1♀).

**Figure 19.**
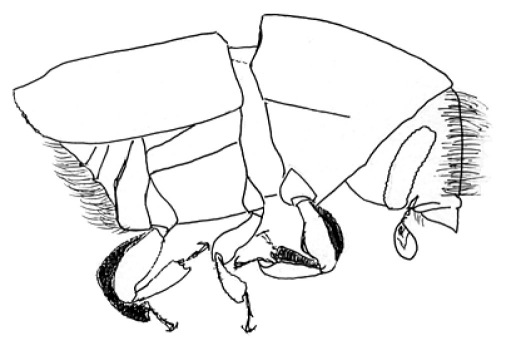
Habitus, lateral view of Scolytus neofacialis

#### Diagnosis.

Species differs from Scolytus bolivianus Schedl by smaller punctures on pronotal disk, by shorter bristles at elytral interstriae and significantly more abundant hairs at female front (the latter species is known only by female holotype).

#### Description.

##### Male:

Body length 3.2–3,4 mm, 2.1 times as long as wide; colour dark brown, elytra reddish brown. Front faintly convex, strongly shining; center of front and epistoma with sparse longitudinal furrows, lateral sides of front densely aciculate from vertex to epistoma. Vestiture on lateral and dorsal pronotal margins of long, moderately abundant, incurved hair, vestiture in central area shorter, less abundant. Antenna brown, covered by short golden hairs, club elliptical, with apex evenly rounded. Pronotum 1.04 times as long as wide, reddish brown, with darker apical margin; surface smooth, shining, punctures small in disk, on lateral margins larger and subrugose on anterior area; lateral margins and anterior area of pronotum covered by thin, short yellow hairs; pronotum divided from prosternite by a well-developed acute lateral margin. Lateral sides of prosternite (propleura) roughly punctured by punctures of moderate size, these punctures smaller compared to punctures at lateral sides of pronotum.

Scutellum triangular, not deeply set in scutellar impression.

Elytra reddish brown, unicolorous, surface faintly shining, nearly dull. Elytra 1.2 times as long as wide, 1.2 times as long as pronotum, striae weakly impressed in base of elytra and narrowly, distinctly impressed on posterior half; interstriae on basal portion flat, smooth, not impressed, in posterior half impressed equally to striae, bristle-carrying interstrial punctures larger compared to strial punctures, interstriae with rows of numerous erect golden bristles organized on the complete elytral surface in regular rows. Abdomen reddish brown, its surface faintly shining, nearly dull; second sternite subvertical, junction with first sternite abrupt, transversely subcostate, fifth sternite weakly concave, apical margin weakly elevated. All sternites abundantly covered by long yellow hairs, curved towards elytral apex.

##### Female:

body length 3.2 mm, 2.1 times as long as wide; colour reddish brown, pronotum 1.04 times as long as wide, elytra 1.0 times as long as wide, 1.0 times as long as pronotum, morphology similar to male, except front and abdomen. Front convex with yellow moderately long hairs, curved towards center of the front, in the middle of the front hairs are shorter when compared to male; from upper frontal portion longer hairs go down, these hairs oriented towards front centre; abdomen more convex compared to male.

**Figure 20.**
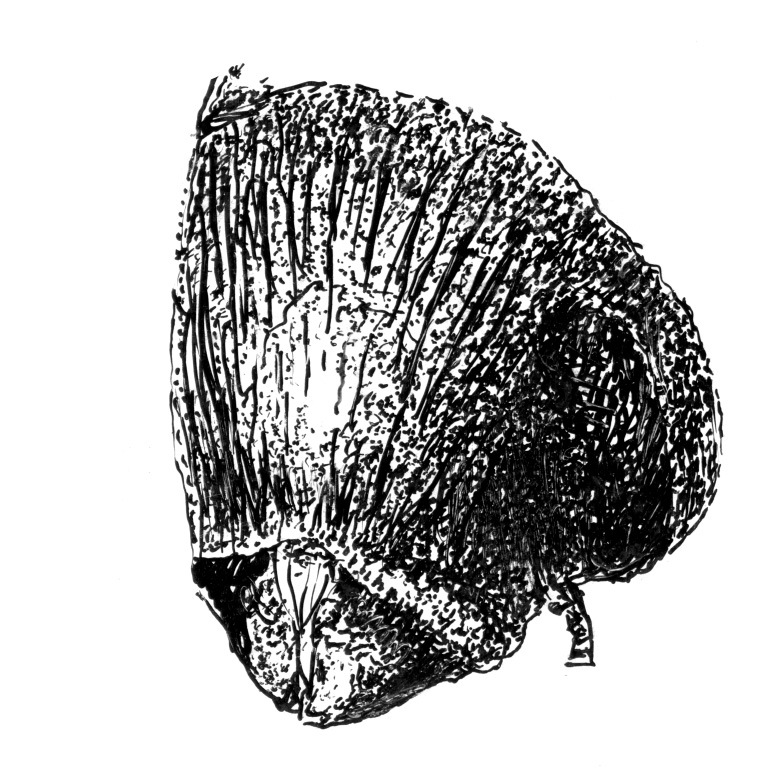
Head of Scolytus neofacialis

#### Notes.

The holotype of Scolytus neofacialis preserved in NHMW (Vienna) is a male. The female is here described for the first time.

The species is very similar to Scolytus bolivianus Schedl, from which it differs only by the body size. It is quite probable that Scolytus neofacialis in the future will turn out to be a synonym of Scolytus bolivianus which was described from one incompletely developed male. Further investigation of Scolytus bolivianus will require a new series.

**Figure 21.**
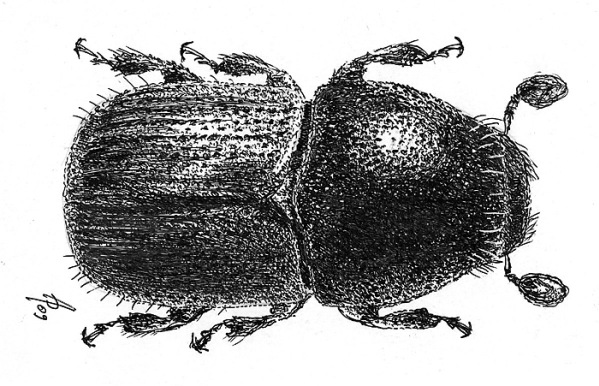
Habitus of Scolytus pinnatus, female

### Scolytus peruensis

Schedl, 1937

#### Material examined.

Peru: Rio Toro, La Merdet Chanchamayo, NHMW, Wien (Lectotype ♂ and paratype ♀)

#### Diagnosis.

Species can be distinguished from Scolytus barinensis Wood by less strongly developed median frontal tubercle in males and also by significantly stronger developed frontal vestiture in males; by more gently punctured pronotum and by quite different elytral sculpture.

#### Description.

##### Male:

body length 3.2–3.4 mm, 1.9 times as long as wide; colour dark reddish brown, pronotum dark brown or black. Front with transverse impression above epistoma and with a conspicuous median tubercle above this impression. Front on upper two–thirds to vertex obscurely aciculate. Front covered by brown hairs more abundant at lateral sides. Antennal funculus and scapus reddish brown. Club grayish brown, ellipsoid in form, evenly rounded at apex and densely covered by short golden hairs. Pronotum 1.0 times as long as wide; lateral sides almost straight and parallel on basal half, arcuately converging toward broadly rounded anterior margin; surface smooth, shining. Pronotal puncturation uneven, punctures of anterolateral angles of pronotum several times larger compared to minute punctures on base and disk.. Apical portion of prontoum with few hairs.

Scutellum triangular, deeply set in scutellar impression.

Elytra 0.9 times as long as wide, 0.9 times as long as pronotum, elytral surface punctured, punctures forming rectilinear weakly impressed rows, punctures in striae of moderate size, not confluent, but rather set apart from their neighbors; interstriae almost four times as wide as striae, smooth, shining, punctures mostly minute; elytral apex with rows of short and sparse erect hairs; abdomen dark reddish brown, surface dull; second sternite subvertical, junction with first sternite abrupt, anterior margin not carinate, its surface rough, with sparse points of moderate size the posterior margin of second sternite; median short laterally compressed tubercle in center of second sternite; third – fifth sternites without denticles and tubercles, punctures at these sternites very small, moderately close. First sternite is covered by short recumbent hairs, vestiture of second – fifth sternites entirely abraded on type. Legs reddish brown, covered by short hairs.

##### Female:

similar to male except details of front and abdomen. Female front more convex than male front, hairs on lateral sides of front shorter; two forelocks consisting of densely set dark brown hairs run from the vertex towards the centre of head. Median spine on second sternite longer then wide, its apex narrowly rounded, spine apex is directed downwards from centre of sternite to its base.

#### Notes:

The above treatment was based on the male (Holotype) and female (Paratype). It is possible that the holotype had traumas immediately after pupation, because its body morphology has numerous evident deviations including strongly shortened elytra with the abnormally curved apex and a shortened second sternite tubercle when compared to female. To confirm the species diagnosis, additional male specimens are required which were lacking at the time of our investigation.

### Scolytus proximus

Chapuis, 1869

Figs 22 23 

#### Material examined.

Peru: Loreto province, 20 km NE from Iquitos, Momon river, Gen Gen vill., 120 m a.s.l., 5–7.02.2007 A.Petrov (48♂♂, 24♀♀); 70 km SW from Iquitos to Nauta, 26–29.02.2008 A.Petrov (41♂♂, 37♀♀ ); 58 km SW from Iquitos to Nauta, Itaya river, 120 m a.s.l., 5–8.V.2009 A.Petrov (4♂♂, 2♀♀).

**Figure 22.**
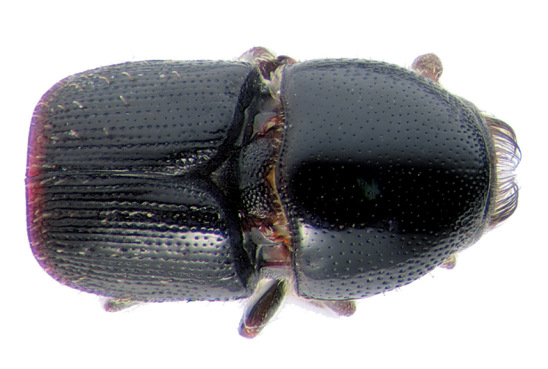
Habitus of Scolytus proximus, male

**Figure 23.**
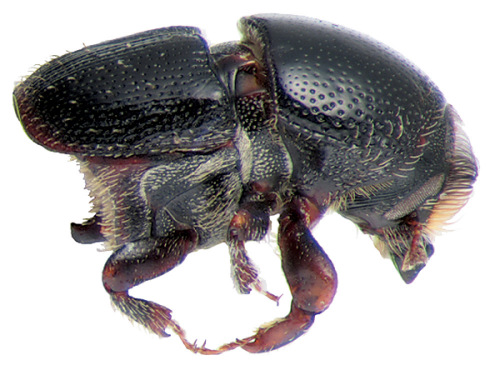
Habitus, lateral view of Scolytus proximus, male

#### Diagnosis.

Species differ from other species in genus by frontal vestiture in male and in female, by elytral puncturation and by form of second abdominal sternite spine that is alike in form to a shark dorsal fin.

#### Description.

##### Male:

body length 2.8–3.5 mm, 1.8–1.9 times as long as wide; colour dark brown black, surface shining. Front flattened on dorsal half, weakly transversely impressed in lower third, lateral areas on lower third somewhat aciculate-granulate, median line on impressed area is slightly elevated. Centre of front with callous-like tubercle slightly rising above impression; above tubercle frontal surface coarsely aciculate. Lateral sides of front slightly elevated, covered with dense brushes of long brown hairs, hair apices curved towards centre of front. Centre of frontal area covered by recumbent hairs of moderate size. Antennal funiculus and scapus reddish brown. Club ellipsoid, grayish brown, densely covered with short golden hairs. Pronotum black, 0.8–0.88 times as long as wide, attains maximum width at the middle of its length; surface smooth, shining, puncturation very small and sparse on disk, more dense and of moderate size on apical margin and of large size at lateral sides of pronotum; surface with few unconspicuous hairs, nearly glabrous. Pronotum separated from prosternite (propleura) by well-developed acute lateral margin, lateral portions of prothorax (propleura) densely punctured by large punctures, surface with recumbent hairs.

Scutellum triangular, deeply set into scutellar impression.

Elytra 0.8–0.94 times as long as wide, 0.9–1.0 times as long as pronotum. First and second rows of punctures impressed from base to declivity, others not impressed or feebly impressed in base and evidently impressed from a middle to apical part of elytra. Punctures in striae of moderate size, sometimes size of punctures become larger from base towards posterior part of elytra. Interstriae three times as wide as striae, smooth, shining, weakly impressed in anterior part of elytra, punctured by small punctures, among which larger setiferous pores can be found in posterior elytral portion. Declivity short; first, third, fifth and seventh interstriae with pale sparse, erect, scale-like hairs forming rows from elytral center and up to apical elytral portion with strongly confused (obscure) puncturation; all other interstriae have only 1–2 short bristles nearby margin of declivity. Abdomen dark brown, surface faintly shining, nearly dull, punctured by small punctures, covered with short pale bristles, under bristles abdominal surface with plumose setae. Erect bristles on second sternite three times as long as on fifth sternite. Second sternite is vertical, with large laterally compressed spine; form of the spine is similar to a shark dorsal fin with its apex curved upwards towards third sternite; size and form of spine is strongly variable between specimens. Legs reddish brown, covered by short yellow hairs.

##### Female:

similar to male except front has shorter hairs evenly covering frontal surface from middle to lateral margins, hairs in centre of front pale, on upper portion of front above upper level of eyes of front a dark fringe consisting of two bundles of dark-grayish brown bristle-like hairs running downwards; hair apices in fringe directed towards front centre.

#### Notes.

Unfortunately, the authors were unable to study the type series of Scolytus proximus and for material determination we used only the original description (Chapuis, 1869) and specimens from the collection of K. Schedl.

#### Biology.

Scolytus proximus infests trunks and large branches of the fallen trees. Parental tunnels are biramous and transverse; the larval mines are longitudinal. Length of transverse tunnels is 20–35 mm.

### Scolytus thoracicus

Chapuis, 1869

#### Material examined.

Peru: Loreto province, 20 km NNE from Iquitos, Momon river, Gen Gen vill., 8.02.2007 A.Petrov

#### Diagnosis.

Species differs from its relatives by male frontal characters and by stepped median spine on sternite 2 in both sexes.

#### Description.

##### Male:

body length 3.0–4.3 mm, 1.9 times as long as wide; colour dark brown or black. Front flattened on dorsal half, strongly, transversely impressed in lower third, impression surface is roughly punctured, median line at impressed portion of front is slightly elevated. Center of front with transverse carina, strongly, dorsoventrally compressed, center of carina strongly thickened and overhanging frontal impression; area above carina smooth and dull, gently shagreen and punctured by sparse minute punctures, devoid of hairs. Lateral parts of front slightly elevated, covered by dense brushes of long brown hairs, hair apices directed towards centre of front. Antennal funiculus and scapus reddish brown. Club grayish brown, of elongate form, 2.1 times as long as wide, abundantly covered by short golden hairs. Pronotum 1.0 times as long as wide, lateral sides almost straight and parallel on basal half, arcuately converging toward broadly rounded anterior margin; surface smooth, shining, punctures minute on disk, much lager on lateral margins of the apical area. Pronotum has an acute lateral margin dividing it from prosternite. Lateral sides of prothorax (propleura) are densely and evenly punctured by punctures that is larger compared to punctures at lateral sides of pronotum, towards apical margin with gentle pale hairs of moderate length.

Scutellum of moderate size, triangular, densely punctured by minute punctures, slightly deepened into scutellar impression.

Elytra 1.0 times as long as wide, 1.0 times as long as pronotum; rows of punctures weakly, narrowly impressed, punctures small, punctures in striae not confluent; interstriae smooth, shining, three times as wide as strial punctures, punctures very small. At the posterior elytral portion among minute punctures of interstriae are larger setiferous pores each bearing one short erect hair. Lateral sides of elytra with a row of yellow hairs of moderate length. Short elytral declivity with obscure puncturation. Abdomen black, its surface dull, evenly punctured by deep punctures of moderate size; second sternite vertical, armed by a median, laterally compressed spine, occupying sternite portion from its base to centre. Apex of tubercle with asymmetrically bifurcated apex, two-stepped. Lateral margins of second sternite with elevated blunt tubercles. Abdominal sternites and spine are covered by erect yellow hairs; longer hairs are located on border between first and second sternites, on lateral sides of sternites and on apex of fifth sternite. Legs dark grayish brown, femora covered with long brown hairs.

##### Female:

similar to male except front convex, without tubercle; punctures fine, surface coarse aciculate; vestiture of fine, uniformly distributed hairs, extending upper level of eyes; spine in second sternite two-stepped as in male but much smaller.

#### Notes.

The species is recorded for Peru for the first time. The male studied did not differ from male of Scolytus thoracicus from Brazil.

### Scolytus vagabundus sp. n.

urn:lsid:zoobank.org:act:113AFEB7-9AB8-4E8A-8068-E0ECE0A00633

Figs 24 25 

#### Type locality.

Peru, Loreto province, left bank of Amazon River.

#### Type material.

Holotype ♀ (ZMM): PERU: LORETO PROVINCE: left bank of Amazon River, 70 km SSW from Iquitos to Nauta, 130 m a.s.l., 1.03.2008, leg.. A.V. Petrov.

**Figure 24.**
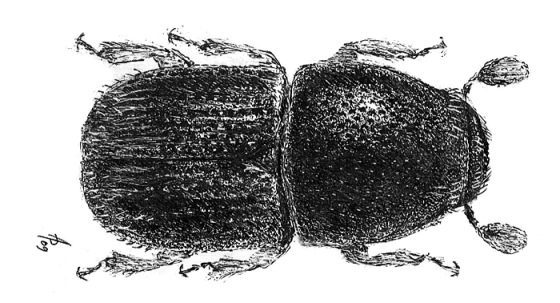
Habitus of Scolytus vagabundus, female

#### Diagnosis.

Species closely related to Scolytus carveli and Scolytus pinnatus Eggers, from which it can be distinguished by body size, by second sternite tubercle form and its position on sternite, by presence of tubercle on fifth sternite and by abdominal vestiture. From Scolytus pinnatus new species can be distinguished also by absence of tubercles and denticles on fourth sternite.

#### Description.

##### Female:

body length 2.2 mm, 2.2 times as long as wide; colour dark brown. Head dark grayish-brown, nearly black. Frons convex from eye to eye from epistoma to vertex; surface aciculate from vertex to epistoma. Vestiture scant, hairs short and sparse, conspicuous in lateral parts of front. Antenna brown, densely covered by short golden hairs, club elliptical with evenly rounded apex. Pronotum 1.1 times as long as wide, its maximal width at the half of its length, from center to apex pronotum seems more elongated. Pronotal surface shining, punctured by deep points at base and in central part of disk; punctures of lateral sides of pronotum and its apical portion slightly larger. Apical portion of pronotum with few short dark hairs. Pronotum has a well defined acute lateral margin separating it from prothorax (propleura). Lateral sides of prothorax (propleura) are abundantly and evenly punctured by punctures of moderate size.

Scutellum triangular, deeply set in scutellar impression.

Elytra dark grayish brown with reddish brown declivity. Elytra 1.1 times as long as wide, 1.0 times as long as pronotum; lateral elytral margins slightly widened from base to middle of their length, these sides are evenly rounded then towards elytral declivity. Elytral surface shining, punctured with sparse punctures in striae. Striae and interstriae are deepened, interstriae with few and sparse punctures, evident only in posterior part of elytra. In the posterior part of elytra and on declivity, interstriae with rows of pale sparse erect hairs. Abdomen reddish brown. Second sternite set vertically, perpendicular to first sternite, anterior margin of second sternite weakly rounded, not costate, sternite two armed by a large, laterally compressed median spine, this spine basis runs through second sternite from its base and up to posterior margin. On dorsum of the spine a sharpened denticle directed by its apex downwards; this denticle located closer to base of second sternite. Lateral sides of second and third sternites with small sharpened denticles. Fifth sternite with a median tubercle. Surface of second sternite smooth and shining, without puncturation, other sternites with minute dense puncturation. Abdomen densely covered by yellow hairs of moderate length, hair apices directed toward tubercle of fifth sternite. Legs reddish brown, with golden hairs.

##### Male:

Unknown.

**Figure 25.**
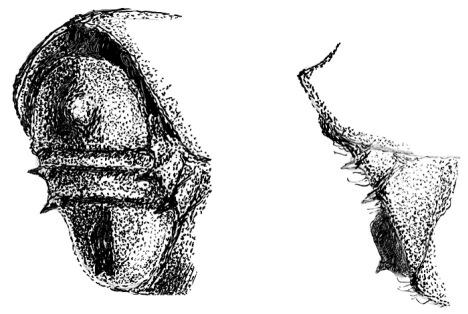
Sternites of abdomen of Scolytus vagabundus, female

#### Distribution.

Known only from the type locality.

#### Etymology.

Species name originates from the Hispan word “vagabundo” (vagabond).

### Scolytus woodi sp. n.

urn:lsid:zoobank.org:act:99DBFD74-8414-4F8A-B3A7-B89FDC4A605B

Figs 26 31 

#### Type locality.

Peru, Loreto province, left bank of Amazon River, Itaya River.

#### Type material.

Holotype ♂ (ZMM): PERU: LORETO PROVINCE: right bank of Amazon River, 30 km S from Iquitos, Panquana camp., 120 m a.s.l., 30.01.1997, leg.. A.V. Petrov. **Paratype**: 1♀ (Petrov collection): PERU: LORETO PROVINCE: Itaya river, left bank of Amazon River, 58 km SSW from Iquitos to Nauta, 120 m a.s.l., 4°11'S; 73°26'W 10.02.2007, leg.. A.V. Petrov (1♀).

**Figure 26.**
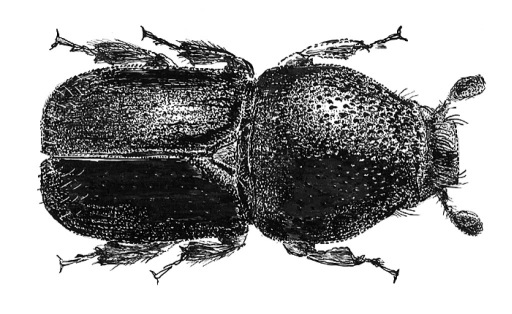
Habitus of Scolytus woodi, male

**Figure 27.**
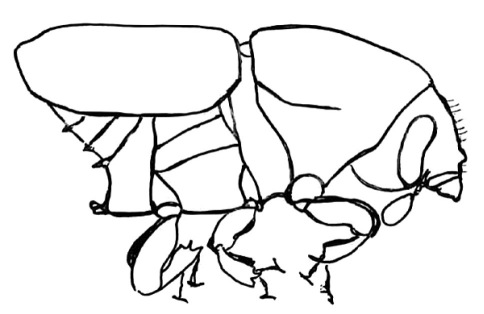
Habitus, lateral view of Scolytus woodi, male

#### Diagnosis.

Species morphologically closely related to Scolytus bispinatus and Scolytus carveli, from which can be distinguished with ease by form and position of second sternite tubercle and also by presence both in male and in female of small sharpened median tubercle at posterior margin of fourth sternite.

#### Description.

##### Male:

Body length 2.0 mm, 2.85 times as long as wide; colour reddish brown. Head dark grayish brown. Front flat with a small median elongate tubercle running from epistoma nearly up to center of front. Circular impression clearly seen in upper portion of front above upper level of eyes nearby margin with vertex. Frontal surface shining, longitudinally aciculate. Lateral parts of front covered by very sparse grey hairs of moderate length, their apices directed towards center of front. Antennae brown, covered by short golden hairs, club elliptical with evenly rounded apex. Pronotum 1.0 times as long as wide. Maximal width of pronotum at half of its length, lateral sides evenly rounded towards apex and base; towards apex, pronotum appears more elongate. Faintly elevated median line runs from centre of pronotum towards its apical portion. Pronotum grayish brown, its basis lighter, reddish-brown; surface shining, at basis and in central part of disk pronotum with small shallow punctures, punctures at lateral sides and in apical portion larger but also shallow. Sharply elevated lateral margin separates pronotum from other parts of prothorax (propleura). Lateral sides of prothorax (propleura) are abundantly and evenly punctured by punctures of moderate size. Scant pubescence limited to few hairs at apical portion of pronotum.

Scutellum triangular, deeply set in scutellar impression.

**Figure 28.**
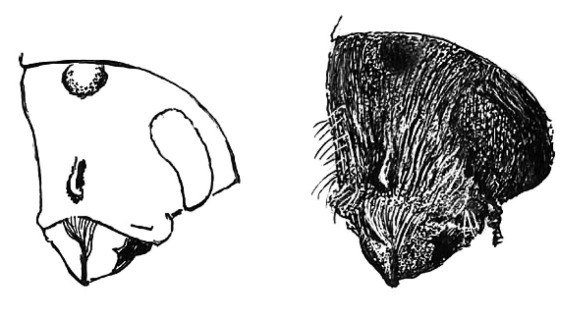
Head of Scolytus woodi, male

**Figure 29.**
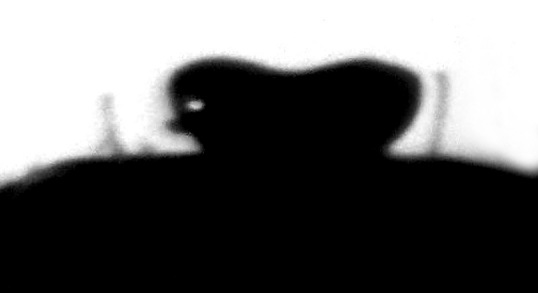
The central spine of second sternit of Scolytus woodi, male

Elytra reddish-brown. Elytra 1.1 times as long as wide, 1.0 times as long as pronotum; lateral sides of elytra are nearly parallel up to declivity, from the beginning of declivity and up to sutural apex elytra are evenly rounded. Elytral surface shining, with regular rows of small punctures. Interstriae flat and smooth, with sparse punctures, conspicuous only in posterior portion of elytra. In posterior portion of elytra, interstriae with rows of pale erect hairs. Abdomen reddish-brown. Second sternite is vertical, perpendicularly set in relation to first sternite, anterior margin of second sternite weakly elevated, costate. Second sternite base armed by a large, median spine. This spine has a very specific outline, its two rounded apices are directed into opposite sides from the same basis, so when looking from below tubercle has form of stylized heart (Fig. 28). Elevated median line runs from base of tubercle towards second sternite center. Posterior margin of fourth sternite with small median sharpened tubercle(Fig. 29). Lateral sides of second and third sternites with minute denticles, second sternite with sharpened denticles, third sternite with smaller denticles with blunt apices. Abdominal surface shining, punctured by minute punctures, covered with very sparse tiny erect hairs. Legs reddish brown, with golden hairs.

##### Female:

similar to male except front more convex, vestiture less abundant and shorter, tubercle in anterior frontal portion of triangular form; second sternite unarmed. As in male, posterior margin of fourth sternite with small median sharpened tubercle at posterior margin, lateral sides of second and third sternites with denticles, but these denticles are extremely small.

**Figure 30.**
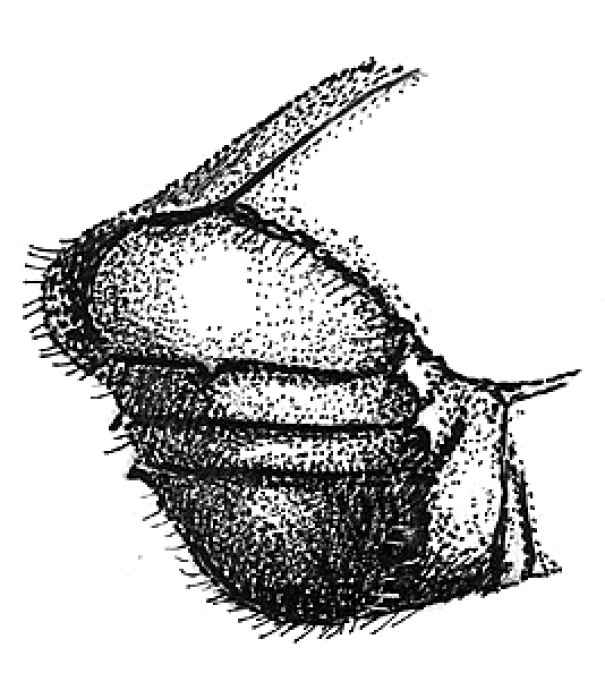
Sternites of abdomen of Scolytus woodi, female

**Figure 31.**
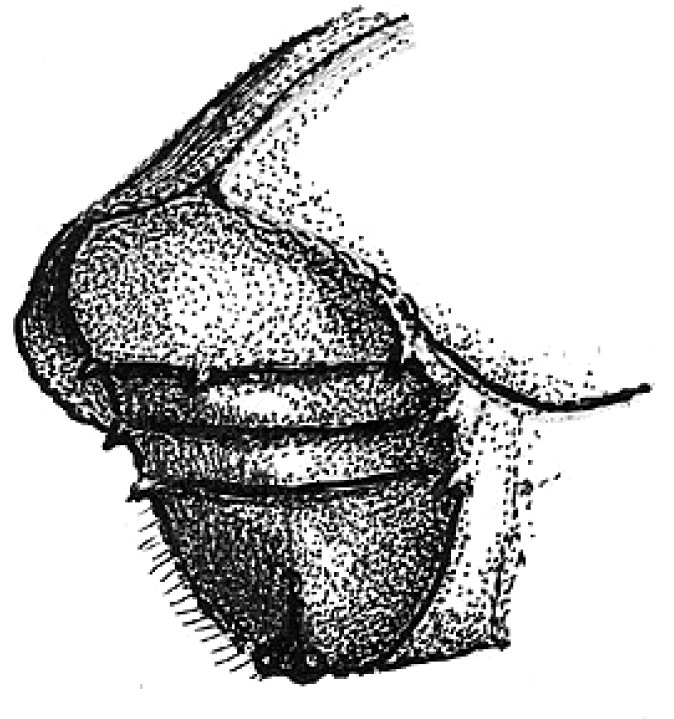
Sternites of abdomen of Scolytus woodi, male

#### Distribution.

Known only from the type locality.

#### Etymology.

This new species is named in honour of the eminent entomologist Professor Stephen L. Wood who dedicated his life to studies of Scolytidae and Platypodidae.

## Key to Peruvian Scolytus Geoffroy, 1762 species

**Table:** 

1.	Front without median tubercle or longitudinal carina	2
–	Front with median tubercle or longitudinal carina	17
2(1)	Antennal club very strongly elongated, its length is more than 2.5 times greater than its width and more than 3.0 times greater than the combined length of scapus and funiculus (Fig. 5); central portion of elytra with oblique central light band; in males second sternite not armed, fifth sternite with large median denticle; in females second sternite is armed by laterally compressed spine and fifth sternite has no denticles and tubercles; 1.9 –3.1 mm; Brazil and Peru	Scolytus antennatus Schedl
–	Ratio between antennal length and width does not exceed 2.0; elytra without light oblique band	3
3(2)	Frontal surface aciculate	4
–	Frontal surface is punctured or upper and central portions of front are transversely rugose	13
4(3)	Scutellum is reduced, invisible when viewed from above; antennal club with narrowed and pointed apex; form of suture on club resembles Greek letter lambda ; base of pronotum with median projection; elytra are covered by short erect dark setae from base and up to apex; in male second sternite subvertical relative to first sternite, its anterior margin subacutely costate on median area, in female second sternite with costa absent, its base rounded, anterior margin with small bifurcated callus; 3.2–4.1 mm; Costa Rica to Brazil	Scolytus costellatus Chapuis
–	Scutellum normal, triangular in shape; antennal club with its apex rounded; base of pronotum without median projection	5
5(4)	Abdominal sternites with laterally compressed tubercle or with longitudinal carina	6
–	All sternites without median tubercles or longitudinal carina	11
6(5)	Sternites 4 and 5 with median tubercle or callous-like elevation	7
–	Sternites 4 and 5 without median tubercle or callous-like elevation	8
7(6)	Second sternite with median laterally compressed tubercle that occupies distance from sternite base and up to its center; fourth sternite with small sharpened tubercle at its posterior margin. Brazil	Scolytus pinnatus Eggers (Fig. 21)
–	Second sternite with median laterally compressed tubercle, that occupies the distance from the basal area and up to posterior sternite margin, nearby anterior margin of second sternite this tubercle has sharpened projection oriented downwards; fourth sternite has no tubercles; central portion of fifth sternite with high callous-like elevation; 2.2 mm, Peruvian Amazonia	Scolytus vagabundus sp. n.
8(6)	Lateral sides of second and third abdominal sternites without sharp denticles	9
–	Lateral sides of second and third abdominal sternites with sharp denticles	10
9(8)	Median carina of second abdominal sternite is rounded at its apex; striae and interstriae 1 and 2 impressed from base of elytra to declivity; 2.3–3.3 mm; Mexico to Peru	Scolytus cristatus Wood
–	Denticle at the second sternite is not rounded at apex, but longitudinally bifurcate (median spine stepped on sternite 2) 3.0–4.2 mm; Brazil to Peru	Scolytus thoracicus Chapuis (female)
10(9)	Median tubercle on second abdominal sternite is of rectangular form, its height not greater than the half of tubercle length; elytral puncture rows are not deepened at base of elytra, in posterior third of elytral length rows of punctures are only slightly deepened, interstriae flat and smooth with only sparse punctures visible only in posterior portion of the elytra; laterally set tubercles of second sternite are evidently sharpened both in male and in female; female without median tubercle at second sternite; 1.9–2.1 mm Peruvian Amazonia	Scolytus carveli sp. n.
–	Median tubercle of second abdominal sternite is laterally compressed, higher than basal width, its apex slightly curved dorsad; third and fourth abdominal sternites with very small tubercles; 2.0 mm; Brazil	Scolytus elongatus Schedl
11(5)	Lateral sides of second abdominal sternite without tubercles; the surface of second sternite shiny, covered from center and up to posterior margin by deep large punctures; base of second sternite with few sparse punctures, shining; base of antennal club is significantly narrower compared to its apical portion; in male lateral portions of front are covered by long dark hairs curving towards frontal centre, in female front is convex with short hairs, vertex without fringe; 2.7–3.3 mm; Peruvian Amazonia	Scolytus mozolevskae sp.n.
–	Lateral sides of second abdominal sternite with small tubercles	12
12(9)	Surface of second abdominal sternite is evenly covered with punctures of middle size, abdominal surface is evenly covered by dense yellow hairs; entire elytral surface is covered with rows of short pale hairs on interstriae; width of the antennal club at base and in apical portion is nearly the same. Male front is covered by the abundant long hairs, in female hairs in the central portion of front are shorter, from the vertex originates a fringe of longer but rather sparse hairs; 3.2–3,4 mm, Brazil and Peru (Amazonia)	Scolytus neofacialis Schedl
–	Puncturation of second abdominal sternite is uneven, present only locally, punctures of second sternite are grouped only at distinct slightly deepened areas; around these areas is surface dull, slightly elevated, with no puncturation, vestiture sparse, only locally developed; elytral interstriae with rows of sparse hairs developed only posteriorly ; base of antennal club is significantly narrower compared to its apical portion; in male lateral portions of front are evenly rounded and covered by long golden hairs, forming a brush; in female brush in center of front is shorter but the fringe of reddish-brown hairs originating from the vertex overhang upper portion of front; 4.0–4.5 mm; Brazil, Peru (Amazonia)	Scolytus amazonicus Schedl
13(3)	Front deeply transversely concave from eye to eye and longitudinally from epistoma to above upper level of eyes; base of each mandible with process (in older beetles these processes may be broken off); scutellum is very small; second abdominal sternite with the median long cylindrical tubercle which is thickened apically; 4.8–5.3mm; Peru to Bolivia	Scolytus excavatus Wood (female)
–	Front faintly or distinctly convex, mandibles without processes, scutellum of normal size	14
14(13)	Lateral sides of second abdominal sternites without sharpened tubercles; front with slightly elevated median line from epistoma up to center of front; from centre of frontal flat, shining, slightly impressed in its centre, area runs toward vertex; two orange fringes overhang this area symmetrically on both lateral sides; this fringes are formed by long hairs; hairs at lateral sides of front are short, with their apices directed towards centre of front; second sternite subvertical, its junction with first sternite abrupt; 2.2–3.6 mm; Brazil, Peru (Amazonia)	Scolytus angustatus Browne (female)
–	Lateral sides of the second abdominal sternite with sharpened tubercles	15
15(14)	Second abdomainal sternite without median tubercle, in male central portion of first sternite apical margin and basis of second sternite are slightly projecting backwards; border between first and second sternites is blurred; base of second sternite with two unconspicuous callous-like tubercles, lateral sides of first sternite are narrowed; lateral denticles of second sternites with attenuated apices, conspicuous; in female border between first and second abdominal sternites is faintly rounded, without microscopic callous-like tubercles, lateral denticles of the second sternite are small, front with transverse rugosities above frontal center and up to vertex; 1.5–1.8 mm; Brazil to Peru	Scolytus bicinctus Schedl
–	Second abdominal sternite with small tubercle near posterior margin	16
16(15)	Second sternite without sharpened denticles on basal margin and on lateral sides of posterior margin; junction of first and second sternites is rounded; frontal surface with moderately long white hairs; 2.2 mm; Surinam	Scolytus spinidens Schedl
–	Lateral sides of second abdominal sternite basal margin with two small sharpened tubercles, lateral sides of second sternite posterior margin also with pair of sharpened tubercles; front is gently covered with small shallow punctures, frontal hairs short and sparse; 2.0 mm; Peru (Amazonia)	Scolytus lindemani sp. n., female
17(1)	Second sternite without median tubercle. Front is covered by small punctures; in male first abdominal sternite apex and second sternite basal area projecting backwards in the centre, anterior margin of second sternite very strongly carinate on median third of segment width, or lateral profile of abdomen is incurved from second sternite and up to fifth sternite apex; in female second sternite subvertical, without central curvature; in male lateral parts of second and third sternites with sharpened tubercles, in females these tubercles nearly indistinct; male front faintly convex, flat above frontal tubercle, covered by moderately long hairs; in female front with median elevated line, longitudinal impression in upper portion of front and with two orange fringes overhanging front from vertex; 2.2–3.6 mm; Brazil, Peru (Amazonia)	Scolytus angustatus Browne
–	Second sternite with median tubercle	18
18(17)	Front impressed from epistoma up to the upper level of front; lateral frontal surface gently punctured; frontal margin that is adjacent to mandibles is strongly incurved towards center of the front; center of the front with circular impression; mandibles with deep transverse furrows and rugosities; base of mandibles with blunt elevated tubercles; second abdominal sternite with a tubercle in a form of a shark back fin curved upwards; 5.5–6.4 mm; Bolivia to Peru)	Scolytus excavatus Wood (male)
–	Front not impressed, flat or convex; in the case of faint circular impression presence it is developed only on the upper portion of front at the border with vertex; front longitudinally aciculated, mandibles without transverse furrows and processes	19
19(18)	Posterior margin of fourth sternite with sharpened tubercle; lateral sides of second and third sternites carry tubercles that are of median size and with sharpened apices in male, minute and blunt-ended in female; second sternite base in male with tubercle bifurcated at apex, second sternite in female without tubercle; front in male with impression at the border between upper portion of front and vertex; 2.0–2.1 mm; Peru (Amazonia)	Scolytus woodi sp. n.
–	Posterior margin of fourth abdominal sternite without tubercle; head in male without impression at border between front and vertex	20
20(19)	Median tubercle is located near center or in posterior portion of second sternite, this tubercle is not laterally compressed	21
–	Median tubercle is laterally compressed, it occupies the space from the basal portion of second sternite and up to its centre	22
21(20)	Tubercle is located in the centre of the second abdominal sternite; color dark reddish brown; 2.5 mm; Argentina to Brazil	Scolytus caudatus Eggers
–	Tubercle of the second sternite is very small, apically sharpened, located closer to the posterior sternite margin, but does not touch this margin; color black; 3.0–3.3 mm Brazil to Peru	Scolytus canellae Wood (male)
22(20)	Median spine is longitudinally bifurcate at its apex; male front flattened on basal half, rather strongly, transversely impressed on lower third, median tubercle strongly, dorsoventrally compressed; vestiture very long; 3.0–4.2 mm; Brazil to Peru	Scolytus thoracicus Chapuis (male)
–	Median tubercle at second abdominal sternites is not bifurcate; male front without transverse carina	22
22(21)	Apex of tubercle on second sternite is directed upwards; tubercle is shark back fin-shaped; vestiture of abdomen abundant with palmate and simple setae; male frontal hairs at center and at lateral sides are dark brown, in female hairs of front center are significantly paler in colour and there is a fringe above the upper level of eyes directed downwards which is formed by two fascicles of dark brown hair-like setae with their apices directed towards center of front; 2.8–3.5 mm; Venezuela, Colombia, Peru	Scolytus proximus Chapuis
–	Apex of tubercle on second sternite is directed downwards; abdominal hairs short and sparse; in female median tubercle is longer than in male, rounded apically; in female front without fringe; 3.2 – 3.4 mm; Peru	Scolytus peruensis Schedl

## Supplementary Material

XML Treatment for Scolytus amazonicus

XML Treatment for Scolytus angustatus

XML Treatment for Scolytus antennatus

XML Treatment for Scolytus bicinctus

XML Treatment for Scolytus canellae

XML Treatment for Scolytus carveli

XML Treatment for Scolytus costellatus

XML Treatment for Scolytus cristatus

XML Treatment for Scolytus lindemani

XML Treatment for Scolytus excavatus

XML Treatment for Scolytus mozolevskae

XML Treatment for Scolytus neofacialis

XML Treatment for Scolytus peruensis

XML Treatment for Scolytus proximus

XML Treatment for Scolytus thoracicus

XML Treatment for Scolytus vagabundus

XML Treatment for Scolytus woodi
